# Supplementary material for: Assessing international alcohol consumption patterns during isolation from the COVID-19 pandemic using an online survey: highlighting negative emotionality mechanisms
Source: BMJ Open. 2020 Nov 26;10(11):e044276. doi: 10.1136/bmjopen-2020-044276 (PMC7692002; doi:10.1136/bmjopen-2020-044276)
Supplement: Supplementary data [file bmjopen-2020-044276supp001.pdf]

SUPPLEMENTARY MATERIALS

Demographics for drinkers

	Age		Sex		Country		SES		Relationship
Mean	31.4	Male	599	Total	49	Lower	328	Single	449
SD	13.2	Female	248	UK	347	Mid	176	Relationship	408
Range	18-90	Other	12	US	223	Higher	250		
Depression		Anxiety		PTSD		Depression & Anxiety			
41		27		3		35			

Demographic analysis for study dropouts

Although a majority of the dropout subjects (n=1,515) who entered the study provided no data (n=981), we performed a demographic analysis on dropout subjects who provided this information (n=481) to assess if those who completed the survey differed in demographic factors from those who did not. The mean age of dropout subjects was 26.58 ± 11.11 years [CI: 25.5927.58] (range= 18-80 years), significantly younger than the mean age of individuals who completed the survey (U= 3.69, [CI: 1.15-3.54] $p< .0001$). Further, more males (n=387) than females (n=87) or other genders (n=7) dropped out of the study prior to completion (X^2= 61.23, $p< .0001$).

Sub-sample analysis by country

United Kingdom (UK)

In the UK, the change in problem drinking severity (AUDIT-C) was 1.05 ± 1.46 [CI: 0.911.19] (range: 0-8), and the mean change in the amount consumed was 5.93 ± 11.75 [CI: 4.82-7.05], units per week (range: 0-120). Current problem drinking severity (full AUDIT) was 4.09 ± 4.94 [CI: 3.62-4.56] (range: 0-27). Of the subjects who reported they consume alcohol (n=434), the change in severity from pre-quarantine to quarantine was a decrease of -0.16 ± 2.15,

[CI: 0.3-0.06] (range -8-6) but not significantly so (U= -1.38, [CI: 0.01-0.89] *p*= .19). The units of alcohol consumed per week was significantly increased during the quarantine period (11.25 ± 17.73 units, [CI: 9.36-13.13] range= 1-120) compared to November (10.94 ± 14.17 units, [CI: 9.44-12.45] range = 0-150), U= 3.0, [CI: 0-0.7] *p*= .003. Further, 60 (14%) subjects reported abstention from alcohol consumption during the quarantine period. More subjects reported a decrease (n= 151, 43%) or an increase (n= 130, 39%) as opposed to no change (n= 61, 18%) of weekly alcohol consumption from November to the quarantine period (X^2= 7.2, *p* = .007). The Oxford COVID-19 Government Response Tracker [24] indicated that the lockdown stringency index in the UK during data collection (05/12/2020 to 05/28/2020) was 88.89, with 15,684 confirmed cases and 488 deaths.

United States (US)

In the US, change in problem drinking severity (AUDIT-C) was 1.01 ± 1.55 units [CI: 0.85-1.17] (range: 0-8), and the mean change in the amount consumed was 3 ± 5.51 [CI: 2.39-4] units per week (range: 0-34). The current problem drinking severity (full AUDIT) was 3.48 ± 4.95 [CI: 34] (range: 0-32). Of the subjects who reported they consume alcohol (n= 353), the change in severity from pre-quarantine to quarantine was a decrease of -0.11 ± 2.42 [CI: -0.43-0.21], range -8-8 (U= -0.66, [CI: 0.05-0.9] *p*= .51), but not significantly so. The units of alcohol consumed per week increased between the quarantine period (7.39 ± 11.45 units, [CI: 5.88-8.9] range= 080) and November (6.93 ± 9.78 units, [CI: 5.88-8.9] range = 0-96), but not significantly so (U= 1.1, [CI: 0.01-0.94] *p*= .29). Further, 44 (13%) subjects reported abstention from alcohol consumption during the quarantine period. More subjects reported a decrease (n= 90, 41%) or an increase (n= 88, 40%) as opposed to no change (n= 45, 21%) of weekly alcohol consumption from November to the quarantine period (X^2= 8.15, *p* = .004). The Oxford COVID-19 Government Response Tracker [24] indicated that the lockdown stringency index in the US during data collection (05/12/2020 to 05/28/2020) was 70.92, with 1,347,916 confirmed cases and 80,684 deaths.

Canada

In Canada, change in problem drinking severity (AUDIT-C) was 0.67 ± 1.45 [CI: 0.31-1.03]

(range: 0-8), and the mean change in the amount consumed was 3.03 ± 7.45 [CI: 1.17-4.89] units per week (range: 0-49). The current problem drinking severity (full AUDIT) was 2.78 ± 4.24 [CI: 1.7-3.85] (range: 0-24). Of the subjects who reported they consume alcohol (n= 35), the change in severity from pre-quarantine to quarantine was an increase of 0.16 ± 2.2, [CI: -0.620.95](range= -8-5), but not significantly so (U= .77, [CI: 0.03-0.98] *p*= .44). The units of alcohol consumed per week was decreased during the quarantine period (8.03 ± 14.22 units, [CI:] range= 0-50) and November (6.71 ± 9.49 units, [CI: 3.46-9.97] range = 0-25), although not significantly so (U= 0.17, [CI: 0.59-1.0] *p*= .86). Further, 4 (12%) subjects reported abstention from alcohol consumption during the quarantine period. More subjects reported an increase (n= 16, 46%) as opposed to a decrease (n= 10, 29%) or no change (n= 9, 26%) of weekly alcohol consumption from November to the quarantine period, although not significantly so (X^2= 0.03, *p*= .85). The Oxford COVID-19 Government Response Tracker [24] indicated that the lockdown stringency index in Canada during data collection (05/12/2020 to 05/28/2020) was 70.83, with 69,981 confirmed cases and 4,993 deaths.

Sub-sample analysis by gender

Males

For the males in our sample (n=1,000), the change in problem drinking severity (AUDIT-C) was in 0.91 ± 1.53 [CI: 0.82-1.01] (range: 0-8) and the mean change in the amount consumed was 3.88 ± 8.84 [CI: 3.33-4.42] units per week (range: 0-120). The current problem drinking severity (full AUDIT) was 2.99 ± 4.61 [CI: 2.71-3.28] (range: 0-32), with 403 males included that do not consume alcohol. Of males who reported they consume alcohol (n= 597), the change in severity from pre-quarantine to quarantine was a decrease of -0.4 ± 2.4, [CI: -0.5- -0.21] range -8-8 (U= 3.57, [CI: 0.0-0.03] *p*< .0001). The units of alcohol consumed per week was significantly decreased during the quarantine period (8.52 ± 14 units, [CI: 7.33-9.71] range= 0-120) compared to November (9.23 ± 12.62 units, [CI: 8.21-10.24] range = 0-120), U= -5.2, [CI: 0.0-0.13] *p*< .0001. Further, 128 (20%) males reported abstention from alcohol consumption during the quarantine period. More males reported a decrease (n= 278, 47%) or an increase (n= 204, 34%) as opposed to no change (n= 115, 19%) of weekly alcohol consumption from November to the quarantine period (X^2= 15.94, *p* < .0001).

Females

For females in our sample (n=342), the change in problem drinking severity (AUDIT-C) was 0.81 ± 1.1 [CI: 0.69-0.92] (range: 0-8) and the mean change in the amount consumed was 2.82 ± 4.6 [CI: 2.31-3.32] units per week (range: 0-38). The current problem drinking severity (full AUDIT) was 3.14 ± 4.47 [CI: 3.13-4] (range: 0-21), with 95 females included that do not consume alcohol. Of females who reported they consume alcohol (n= 247), the change in severity from pre-quarantine to quarantine was an increase of 0.12 ± 1.6, [CI: -0.08-0.32] range 5-8, although not significantly so (U= 1.17, [CI: 0.01-0.93] *p*= .24). The units of alcohol consumed per week was decreased during the quarantine period (6.94 ± 10.62 units, [CI:] range= 0-80) compared to November (6.01 ± 8.08 units, [CI: 5-7.02] range = 0-90), although not significantly so (U= -0.57, [CI: 0.1-0.99] *p*= .57). Further, 43 (17%) females reported abstention from alcohol consumption during the quarantine period. More females reported a decrease (n= 102, 41%) or an increase (n= 101, 41%) as opposed to no change (n= 44, 18%) of weekly alcohol consumption from November to the quarantine period (X^2= 13.46, *p* = .0002).

Sub-sample analysis by age

In terms of overall current drinking severity during quarantine, older individuals (5.9 ± 4.82, [CI: 5.44-6.36], range 1-28) showed significantly more problem drinking behaviours than younger individuals (4.74 ± 4.46, [CI: 4.32-5.17], range 1-32); (U= 4.94, [CI: 0.00-0.00], *p*< .0001). The older individuals (≥ 25.1 years) in the sample (n=432) also showed a significant increase in drinking severity from November (4.02 ± 2.13, [CI: 3.82-4.23], range 1-13) to quarantine (4.18 ± 2.35, [CI: 4-4.23], range 1-13); (U= 2.07, [CI: 0.05-0.98], *p*= .04). Further, the mean number of units of alcohol consumed between November (9.93 ± 14.24, [CI: 8.58-11.27], range 0-150) and the quarantine period (10.91 ± 16.96, [CI: 9.31-12.52], range 0-120) was significantly increased (U= 2.75, [CI: 0.01-0.84], *p*= .006).
