# Supplementary material for: Assessing international alcohol consumption patterns during isolation from the COVID-19 pandemic using an online survey: highlighting negative emotionality mechanisms
Source: BMJ Open. 2020 Nov 26;10(11):e044276. doi: 10.1136/bmjopen-2020-044276 (PMC7692002; doi:10.1136/bmjopen-2020-044276)
Supplement: Author's manuscript [file bmjopen-2020-044276.draft_revisions.pdf]

BMJ Open is committed to open peer review. As part of this commitment we make the peer review history of every article we publish publicly available.

When an article is published we post the peer reviewers' comments and the authors' responses online. We also post the versions of the paper that were used during peer review. These are the versions that the peer review comments apply to.

The versions of the paper that follow are the versions that were submitted during the peer review process. They are not the versions of record or the final published versions. They should not be cited or distributed as the published version of this manuscript.

BMJ Open is an open access journal and the full, final, typeset and author-corrected version of record of the manuscript is available on our site with no access controls, subscription charges or pay-per-view fees (http://bmjopen.bmj.com).

If you have any questions on BMJ Open's open peer review process please email info.bmjopen@bmj.com

BMJ Open

Alcohol Consumption Patterns During Isolation from the COVID-19 Pandemic: Highlighting Negative Emotionality Mechanisms

Journal:	*BMJ Open*
Manuscript ID	bmjopen-2020-044276
Article Type:	Original research
Date Submitted by the Author:	28-Aug-2020

Complete List of Authors:	Sallie, Samantha; University of Cambridge, Psychiatry<br>Ritou, Valentin; University of Paris, Faculty of Basic and Biomedical Sciences<br>Bowden-Jones, Henrietta; University College London, Faculty of Brain Sciences; University of Cambridge, Psychiatry<br>Voon, Valerie; Cambridge University, Psychiatry

Keywords:	COVID-19, PSYCHIATRY, Substance misuse < PSYCHIATRY, PUBLIC HEALTH, Depression & mood disorders < PSYCHIATRY

SCHOLARONE™
Manuscripts

I, the Submitting Author has the right to grant and does grant on behalf of all authors of the Work (as defined in the below author licence), an exclusive licence and/or a non-exclusive licence for contributions from authors who are: i) UK Crown employees; ii) where BMJ has agreed a CC-BY licence shall apply, and/or iii) in accordance with the terms applicable for US Federal Government officers or employees acting as part of their official duties; on a worldwide, perpetual, irrevocable, royalty-free basis to BMJ Publishing Group Ltd ("BMJ") its licensees and where the relevant Journal is co-owned by BMJ to the co-owners of the Journal, to publish the Work in this journal and any other BMJ products and to exploit all rights, as set out in our licence.

The Submitting Author accepts and understands that any supply made under these terms is made by BMJ to the Submitting Author unless you are acting as an employee on behalf of your employer or a postgraduate student of an affiliated institution which is paying any applicable article publishing charge ("APC") for Open Access articles. Where the Submitting Author wishes to make the Work available on an Open Access basis (and intends to pay the relevant APC), the terms of reuse of such Open Access shall be governed by a Creative Commons licence – details of these licences and which Creative Commons licence will apply to this Work are set out in our licence referred to above.

Other than as permitted in any relevant BMJ Author's Self Archiving Policies, I confirm this Work has not been accepted for publication elsewhere, is not being considered for publication elsewhere and does not duplicate material already published. I confirm all authors consent to publication of this Work and authorise the granting of this licence.

For peer review only

Alcohol Consumption Patterns During Isolation from the COVID-19 Pandemic: Highlighting Negative Emotionality Mechanisms

Samantha N. Sallie, MS[a]; Valentin JE. Ritou[a,b]; Henrietta Bowden-Jones, MD[a,c]; Valerie Voon, MD, PhD[a]

[a] Department of Psychiatry, University of Cambridge, Level E4, Addenbrooke's Hospital, Cambridge, United Kingdom, CB2 0QQ.

[b] Faculty of Basic and Biomedical Sciences, University of Paris (Université de Paris), 45 rue des Saints-Pères, Paris, France, 75006.

g

[c] Faculty of Brain Sciences, University College London (UCL), London, United Kingdom, WC1E 6BS.

Corresponding author: Samantha N. Sallie, MS, Department of Psychiatry, University of Cambridge, Level E4, Addenbrooke's Hospital, Cambridge, United Kingdom, CB2 0QQ, sns36@cam.ac.uk

Word Count: 4,305

For peer review only

ABSTRACT

Objectives: The Coronavirus (COVID-19) pandemic has required drastic safety measures to control virus spread, including an extended period of self-isolation. Stressful situations increase alcohol craving and consumption in both Alcohol Use Disorder (AUD) and non-AUD drinkers. Thus, we assessed how COVID-19-related stress may have affected drinking behaviours in the general population.

Design: We developed an online cross-sectional survey, Habit Tracker (HabiT), which measured changes in drinking behaviours before (post-hoc recall) and during the COVID-19 quarantine period. We also assessed psychiatric factors such as anxiety, depression, and impulsivity. Lastly, we related drinking behaviours to COVID-19-specific stress factors.

Setting: HabiT was released internationally with individuals from 83 countries participating; a majority residing in the United Kingdom and United States.

Participants: Participants were included if they were 18 years of age or older, confirmed they were proficient in understanding English, and answered attentional checks correctly. The survey was completed by 2,873 adults with 1,346 usable data.

Primary and Secondary Outcome Measures: Our primary outcome measures were change in amount and severity of drinking behaviours before and during quarantine, and current drinking severity during quarantine. These three measures were related to ten COVID-19-related stress factors and current drinking severity to psychiatric symptomology.

Results*:* Although drinking behaviors decreased overall during quarantine, 36% reported an increase in alcohol use. Those who increased alcohol use during quarantine were older individuals, males, essential workers, individuals with children, those with a personal relationship with someone severely ill from COVID-19, and those with higher depression, anxiety, or positive urgency impulsivity.

Conclusions: Our findings highlight a role for identifying those vulnerable for alcohol misuse during periods of enforced self-isolation and underscore the theoretical mechanism of negative emotionality underlying drinking behaviours driven by stress, depression, and anxiety. Future studies should aim to assess the long-term effects of isolation on drinking behaviours.

Keywords: COVID-19; alcohol use; stress; depression; self-isolation

For peer review only

ARTICLE SUMMARY

Strengths and limitations of this study

- The HabiT study sampled drinking behaviours of a large, diverse population during the COVID-19 pandemic.
- Changes in drinking behaviours were assessed against specific COVID-19-related stress factors.
- Due to the length of the survey (8-10 minutes), we observed a large degree of study dropout.
- Subjects were within varying phases of lockdown during the time of testing.
- The prevalence of diagnosed Alcohol Use Disorder drinkers sampled was low, likely related to sampling issues or under-reporting.

For peer review only

INTRODUCTION

The Coronavirus (COVID-19) pandemic has necessitated drastic safety measures to control the virus spread. These measures included an extended self-isolation period in which individuals were permitted to leave their places of residence only to obtain amenities or engage in essential work. Individuals were not permitted face-to-face contact with anyone who did not reside within their immediate households. In the United Kingdom, these measures were instituted nationally on March 23rd, 2020, with a gradual lifting of restrictions on May 10th, 2020 ending on July 4th, 2020 with locality-specific intermittent reinstatement of these measures. Although a necessary precautionary measure to mitigate the devastating effects of COVID-19 on public health, evidence indicates that protracted periods of self-isolation, especially in the context of stress, may be related to acute and prolonged negative mental health consequences, particularly in individuals already struggling with psychiatric disorders.[1]

Indeed, current clinical reports from individuals in treatment for Substance Abuse Disorder indicate that the stress produced by COVID-19 social isolation measures have triggered greater and more frequent drug or alcohol cravings, subsequently leading to relapse.[2] This observation is relevant to a prominent mechanistic theory of negative emotionality underlying alcohol misuse.[3] The relationship between stress and alcohol consumption is widely recognised and can be observed in an experimental fashion.[4] In subjects with known Alcohol Use Disorder (AUD), stress and experimental manipulations of stress enhance the amount of alcohol consumed [5, 6], alcohol craving [7], problematic drinking behaviours, and likelihood of relapse.[8] Exposure to stress triggers relapse characterised by a re-instantiation of alcohol cravings and alcohol-seeking behaviours.

Increases in alcohol craving and consumption after stress exposure also occur in those without AUD. An increase in alcohol consumption is often used as a coping strategy for both chronic and specific stressful life events in both AUD and non-AUD drinkers.[9] Similarly in both groups, self-reported craving and subjective judgements of alcohol value rise following a stress task [10], and social drinkers consume more alcohol after witnessing a social stressor.[11] These relationships are moderated by age [12], gender, previous alcohol exposure [12], underlying personality traits [13], alcohol expectancies [14], and the pattern of alcohol consumption.[15]

For peer review only

Thus, in response to these exceptional circumstances, we aimed to assess how social isolation measures in the midst of the COVID-19 pandemic may have affected drinking behaviours in the general adult population. We developed an international survey, entitled Habit Tracker (HabiT), which evaluated drinking severity before (post-hoc recall) and during the COVID-19 quarantine period. We hypothesised that changes in amount of alcohol consumption and severity of drinking behaviours may be related to specific COVID-19 related stress factors, as well as demographic and psychiatric factors. Further, we investigated if COVID-19-related stress factors influenced changes in drinking amount, drinking severity, depression, and anxiety before and during quarantine.

METHODS

Recruitment and inclusion criteria

The HabiT survey was a questionnaire that sought to assess the effects of isolation on alcohol, smoking, and internet use. The effects on alcohol use are reported here. Subjects were included if they were 18 years of age or older and confirmed they were proficient in reading and understanding English. HabiT was advertised by University of Cambridge news page on May 11th, 2020, a day before its international release. For the next several days, the survey was disseminated by news agencies throughout the UK (e.g., The Telegraph, BBC Cambridgeshire, News Wise) as well as throughout various University of Cambridge colleges. Further, the survey was posted and shared on personal and public social media sites (i.e., Facebook, Twitter). HabiT was approved by the Cambridge Psychology Research Ethics Committee. All subjects gave informed consent and were not financially compensated for their participation. The data collected was fully anonymized. The survey was created using Qualtrics (Provo, Utah) survey-building platform. The average time to complete the survey was approximately 8-10 minutes and all subjects could participate on either a computer or smart phone device.

Patient and public involvement statement

We did not involve patients or the public in the research design, reporting, or dissemination strategies of this study.

For peer review only - http://bmjopen.bmj.com/site/about/guidelines.xhtml

Demographic information

The demographic information collected were as follows: age, gender, socioeconomic status, intimate relationship status, country and city of residence, and any previous or current diagnosis of a psychiatric or neurological disorder.

Attentional checks

Every major section of the survey contained at least one question which served as an attentional check to ensure subjects were correctly reading and answering survey questions to the best of their ability. The attentional checks were structured to mirror the Likert scaling of each section (e.g., "If you are reading this question, please select 'Strongly Agree.'").

Frequency and severity of alcohol consumption before and during the quarantine period

We first asked subjects if they drank alcohol. If the answer was negative, they proceeded to the next set of questions. If the answer was positive, we assessed the change in the amount and severity of alcohol use as well as the current severity of alcohol use. We asked subjects to report the following behaviours within a typical week in November (i.e. pre-quarantine) and within the last week (i.e. during quarantine): (i) the number of units of alcohol consumed within the last week with examples for the number of units for differing types of alcohol and sizes provided; (ii) the change in severity using a time-scale adaptation of the first three questions of the Alcohol Use Disorders Identification Test (AUDIT-C).[16] Subjects were asked to report how many days in the last week they consumed an alcoholic beverage, how many drinks they consumed on a typical day they were drinking in the last week, and how often they consumed six or more drinks on one occasion in the last week. To assess the current severity of drinking behaviours during quarantine, we used a timescale-adapted version of the full AUDIT [17] which assessed problem drinking behaviours within the last week such as an inability to stop drinking once started, failure to perform responsibilities, feeling guilt or remorse, drinking shortly after waking to ease the adverse physiological effects of drinking the night before, drinking to the point of memory loss, injuring oneself or others due to drinking, and concern from a loved one or medical professional related to the frequency or severity of one's drinking. We used two primary outcome measures:

For peer review only

the change in severity (AUDIT-C) corroborated with the secondary change in amount of drinking (units per week) and current severity (full AUDIT).

COVID-19-related stress scale

We assessed 10 factors which may contribute to COVID-19-related stress using the following questions:

1. Have you been deemed an "essential worker" by your government?
2. Do you work for health care services specifically with individuals who have contracted Coronavirus (COVID-19)? (Sub-question of question 1)
3. Has your employment situation changed due to the Coronavirus (COVID-19) crisis?
4. Has anyone you know personally contracted or have shown symptoms characteristic of Coronavirus (COVID-19)?
5. Has anyone you know personally become severely ill or died due to contracting Coronavirus (COVID-19)?
6. Are you isolated alone?
7. Do you have children?
8. If you have children, are you their only caretaker? (Sub-question of question 7)
9. If you are currently in isolation with others, how would you describe the quality of your relations?
10. How often do you currently go outdoors (for work, essential duties, leisure, etc.)?

Psychiatric measures

Depression and anxiety symptomology were measured using The Hospital Anxiety and Depression Scale (HADS); a brief, validated four-item questionnaire.[18] As a secondary analysis, we assessed impulsivity using the validated Short UPPS-P Impulsive-Behavior Scale (SUPPS-P).[19] This scale provides an overall impulsivity score, as well as five scores corresponding to impulsivity subscales: perseveration, lack of premeditation, sensation-seeking, negative urgency, and positive urgency.

For peer review only

Statistical analysis

Statistical analyses were performed using MATLAB (Version 2020a). All subjects who answered the attentional checks incorrectly, reported highly improbable answers regarding the units of alcohol they consumed weekly (e.g., 1,000 units), did not report their gender, or did not complete the psychiatric questionnaires were excluded from further analysis, leaving a total of 1346 subjects. Drinking severity scores of the sample were non-normally distributed (Shapiro-Wilk, $p < .05$), thus non-parametric tests were used.

We used Mann-Whitney U-tests to compare weekly alcohol unit consumption and alcohol severity before and during quarantine in the full group. Then, we divided subjects into three groups, those who during quarantine either increased, decreased, or did not change their alcohol consumption and performed a Kruskal-Wallis H-test to assess the relative drinking amount to severity indices of these groups.

We then assessed which COVID-19-related stress factors were associated with changes in either amount (alcohol units consumed per week), change in severity (AUDIT-C), current severity (full AUDIT), or current depression and anxiety using the following tests: 1) Mann-Whitney U-Tests to compare negative versus positive responses to the COVID-19 stress items (MW), 2) MANCOVA [20] controlling for gender and age (MAN1), and 3) A second MANCOVA controlling for age, gender, depression, and anxiety symptomology (MAN2). For the MANCOVA tests, variables ‘age,’ ‘depression severity,’ and ‘anxiety severity’ were dichotomised via median split. For the COVID-19 stress primary item comparisons (eight items), we used False Discovery Rate (FDR) to control for multiple comparisons with significance assigned at $p < .05$.[21, 22]

On an exploratory basis, we then used Spearman’s partial correlation to compare the drinking severity indices of subjects who completed the timescale-adapted AUDIT with SUPPS-P and HADS scores to relate drinking severity of the overall sample to psychiatric measures. Lastly, in order to assess possible directional relationships in changes in the severity of drinking behaviors to depression, anxiety, and impulsivity; we performed Spearman’s partial correlations with the psychiatric questionnaires among the three aforementioned groups (i.e., increased, decreased,

For peer review only

and null). For both correlational analyses, we used FDR correction ($p < .05$) for multiple comparisons.

RESULTS

Demographic information

A total of 2,873 subjects participated (data collection: 05/12/2020 to 05/28/2020) of which 1,346 had usable data based on defined criteria. Of these subjects, 859 reported that they drink alcohol. Of the 1346 subjects, the average age was 28.92 ± 10.45 years (range= 18-90) with more males (males: n= 1006; females: n=325; other: n=15) from 85 different countries of residence, with the majority from the United Kingdom (n= 434) and the United States (n= 355). Marital status was as follows: single: n=785; married or committed: n=571; divorced or separated: n=33; widowed: n=4. Socioeconomic status was as follows: <19.9k: n=285; 20-39.9k: n= 273; 20-39.9k: n=244; 40-69.9k: n=241; 70-99.9k: n=141; >100k: n=203; and 232 subjects did not report their incomes. Current psychiatric or neurological diagnoses were as follows: no diagnosis: n=1192; depression: n= 60; anxiety: n= 38, Post-Traumatic Stress Disorder (PTSD): n= 5, comorbid depression and anxiety: n= 46.

Overall changes in drinking frequency and severity before and during quarantine

Of the total sample, the change in problem drinking severity (AUDIT-C) was a decrease in 0.89 ± 1.43 (range: 0-8) and the mean change in the amount consumed was 5.62 ± 9.55 units (range: 0-120). The current problem drinking severity (full AUDIT) was 3.14 ± 4.47 (range: 0-32), with 557 subjects included that do not consume alcohol. Of the subjects who reported they consume alcohol (n= 859), the change in severity from pre-quarantine to quarantine was a decrease of 1.53 ± 1.6, range 0-8 (U= 2.65, p= .008). The units of alcohol consumed per week was significantly decreased during the quarantine period (8.03 ± 14.22 units, range= 1-120) compared to November (8.32 ± 11.92 units, range = 0-150), U= -2.29, p= .02 (Figure 1). More subjects reported a decrease (n= 384, 45%) or an increase (n= 308, 36%) as opposed to no change (n= 166, 19%) of weekly alcohol consumption from November to the quarantine period (X^2= 72.86, p = .001; Figure 1). Of the three groups, those who: 1) increased weekly units consumed during quarantine (7.5 ± 10.5 change in units, range: 1-80), 2) decreased weekly units consumed during quarantine (-6.5 ± 9.5 change in units, range: -.2 - -120), and 3) did not change their weekly unit

For peer review only

consumption, subjects who had increased the units of alcohol consumed during the quarantine period showed significantly higher current drinking severity scores (7.5 ± 5.6, range: 1-32) than those who reported decreases (3.5 ± 3.0, range: 1-21) or no changes (4.8 ± 3.6, range: 1-20) in weekly unit consumption (H= 165.33, $p < .0001$).

[INSERT FIGURE 1 & FIGURE 1 LEGEND HERE]

COVID-19 stress item analysis

The change in amount of drinking was positively correlated with age ($r_s = 0.2$, $p < .0001$), and gender with males (6.44 ± 10.8 units, range: 0-120) showing an increase in drinking behaviours relative to females (3.81 ± 5.18, range: 0-38) or other genders (1.32 ± 1.65, range: 0-5) (H= 8.17, $p = .003$). Changes in drinking severity were also related to both age and gender, with older individuals ($r_s = .2$, $p < .0001$) and males (1.68 ± 1.74, range: 0-8) demonstrating greater changes in their drinking severity than females (1.16 ± 1.12, range: 0-8) and others (1.36 ± 1.29, range: 0-3) (H= 6.02, $p = .05$). Thus, we utilised age and gender as covariates for both MANCOVA analyses. All relevant covariates used in these analyses were dichotomised via median split (age= 25.1 years, depression severity= 2, and anxiety severity= 1).

Primary COVID-19 stress items

The influence of COVID-19 stress items on the change in drinking severity, amounts consumed, and current drinking severity are reported in Tables 1, 2, and 3, respectively. Designated essential workers and those with children showed a greater increase in the amount consumed weekly and drinking severity as well as greater current severity. This remained significant including when controlled for demographic variables (age, gender) and psychiatric symptoms (depression, anxiety). Notably, although subjects with children reported an increase in the number of units of alcohol and severity of alcohol use, they also reported lower levels of depression and anxiety. Knowing an individual personally who was ill or severely ill with Covid-19 showed higher current alcohol drinking severity than those who did not, but with no change from pre- to post-quarantine. A reported change in employment status and isolating alone was associated with greater depression scores, with no differences in drinking behaviours. Isolating with others but reporting a poor relationship was associated with greater depression and anxiety,

For peer review only - http://bmjopen.bmj.com/site/about/guidelines.xhtml

however, the lower drinking behaviours were moderated by age and gender effects. Finally, going outdoors was associated with greater current drinking severity and greater depression and anxiety scores controlling for all variables. Post-hoc tests confirmed that, in cases in which a significant relationship was lost between an item and either changes in drinking frequency or severity due to controlling for age and gender (i.e., MANCOVA 1), age was the sole contributor (Essential worker: $F(1, 533.2)= 7$, $p= .008$; Others ill: $F(1, 879.9)= 52.6$, $p < .0001$; Poor relationship: $F(1, 933.9)= 48.88$, $p < .0001$).

Stress Factor	N Total	Yes M(SD)	N Yes	No M(SD)	N No	MW p-value	MAN1 p-value	MAN2 p-value
Essential worker	1337	0.16(1.9)	241	-0.21(1.6)	1096	0.02*	0.01*	0.01*
Employment	1337	-0.14(1.8)	323	-0.14(1.6)	1014	0.83	0.96	0.92
Others ill	1334	-0.17(1.8)	497	-0.12(1.6)	837	0.75	0.64	0.63
Others severely ill	1336	-0.01(2)	127	-0.15(1.6)	1209	0.35	0.7	0.69
Isolated alone	1325	-0.1(1.9)	168	-0.15(1.6)	1157	0.83	0.85	0.82
Having children	1334	0.34(1.4)	209	-0.23(1.7)	1125	<.0001*	0.005*	0.003*
Poor relationship	1168	-0.3(1.7)	187	-0.13(1.6)	981	0.35	0.7	0.69
Going outdoors	1336	-0.27(1.3)	193	-0.12(1.7)	1143	0.26	0.7	0.69

Table 1. COVID-19 primary stress items relationship with changes in drinking severity (as indexed by the AUDIT-C) from pre-quarantine to quarantine.

Stress Factor	N Total	Yes M(SD)	N Yes	No M(SD)	N No	MW p-value	MAN1 p-value	MAN2 p-value
Essential worker	1337	1.26(12.8)	241	0.45(7.5)	1096	0.0003*	0.07	0.08
Employment	1337	0.17(11.2)	323	0.13(7.8)	1014	0.77	0.95	0.97
Others ill	1334	0.05(7.1)	497	0.2(9.6)	837	0.83	0.95	0.97
Others severely ill	1336	0.06(7.6)	127	0.15(8.9)	1209	0.83	0.95	0.97
Isolated alone	1325	0.05(11.6)	168	0.2(8.2)	1157	0.46	0.95	0.97
Having children	1334	2.02(11.9)	209	0.54(7.9)	1125	<.0001*	0.04*	0.02*
Poor relationship	1168	0.4(6.1)	187	0.19(8.7)	981	0.46	0.95	0.97
Going outdoors	1336	1.23(6.8)	193	0.04(9.0)	1143	0.15	0.47	0.4

Table 2. COVID-19 primary stress items relationship with changes in drinking amount (in units) from pre-quarantine to quarantine.

Stress Factor	N Total	Severity Type	Yes M(SD)	N Yes	N M(SD)	N No	M-W p-value	MAN1 p-value	MAN2 p-value
Essential worker	1337	Drinking	4.42(5.7)	243	2.85(4.1)	1099	<.0001*	0.0005*	0.0005*
		Depression	2.29(1.8)	243	2.44(1.9)	1099	0.43	0.84	
		Anxiety	1.79(1.7)	243	1.94(1.8)	1099	0.42	0.8	
Employment	1337	Drinking	3.46(4.9)	324	3.02(4.3)	1018	0.38	0.08	0.144

For peer review only

change		Depression	2.78(2.0)	324	2.31(1.9)	1018	0.0043*	0.007*	
		Anxiety	2.03(4.5)	324	1.88(1.8)	1018	0.32	0.363	
Others ill	1334	Drinking	3.59(1.9)	499	2.87(4.4)	840	<.0001*	0.1	0.125
		Depression	2.3(1.8)	499	2.47(1.9)	840	0.20	0.83	
		Anxiety	1.9(5.5)	499	1.93(1.9)	840	0.99	0.94	
Others severely ill	1336	Drinking	4.49(2.0)	127	2.99(4.3)	1214	0.001*	0.007*	0.01*
		Depression	2.45(2.0)	127	2.4(1.9)	1214	0.99	0.41	
		Anxiety	1.92(5.8)	127	1.91(1.8)	1214	0.82	0.84	
Isolated alone	1325	Drinking	3.88(2.0)	169	2.98(4.2)	1161	0.42	0.83	0.87
		Depression	3.4(1.9)	169	2.41(1.9)	1161	0.009*	0.04*	
		Anxiety	2.04(5.2)	169	1.9(1.8)	1161	0.43	0.11	
Having children	1334	Drinking	5.17(1.8)	211	2.75(4.2)	1128	< 0001*	0.0003*	<.0001*
		Depression	1.5(1.7)	211	2.58(1.9)	1128	<.0001*	<.0001*	
		Anxiety	1.37(1.7)	211	2.02(1.9)	1128	<.0001*	0.0009*	
Poor relationship	1168	Drinking	2.82(5.1)	187	3.1(4.1)	985	0.01*	0.92	0.87
		Depression	3.57(2.0)	187	2.2(1.8)	985	<.0001*	<.0001*	
		Anxiety	2.79(2.0)	187	1.74(1.8)	985	<.0001*	<.0001*	
Going outdoors	1336	Drinking	3.42(4.5)	1148	1.37(3.4)	193	<.0001*	<.0001*	<.0001*
		Depression	3.18(2.0)	193	2.28(1.9)	1148	<.0001*	<.0001*	
		Anxiety	2.42(2.0)	193	1.83(1.8)	1148	0.0002*	0.0008*	

Table 3. COVID-19 primary stress items relationship with current drinking severity (i.e., full AUDIT), depression, and anxiety from pre-quarantine to quarantine.

Secondary COVID-19 stress items

Two COVID-19 stress items were considered secondary as they represented a subset of a primary item. Working for health care services was associated with a trend towards a greater change in amount of units consumed (F= 3.97, p = .05) and greater severity of current drinking (F= 7.01, p = .007) when controlled for all variables. Being the only caretaker for children was also associated with greater change in drinking severity (U= 2.62 , p = .009) and greater change of amount consumed (U= 2.67, p = .007), but was no longer significant when controlling for age and gender.

Drinking severity during quarantine and correlations with psychiatric measures

Of the individuals who reported drinking alcohol, (n= 769) completed the current drinking severity index (e.g., the adapted-timescale AUDIT). The severity of drinking behaviours was positively related to depression (r_s= .12, p= .004), anxiety (r_s= .12, p= .027), and positive urgency impulsivity (r_s= .12, p= .004), controlled for age and gender. To assess potential directional relationships between current drinking severity during quarantine and psychiatric

measures, we correlated depression, anxiety, and impulsivity with the three drinking groups (i.e., increased, decreased, null). Drinking severity scores in the decreased and no change groups were not significantly correlated with any of the psychiatric measures of interest. However, drinking severity of those who increased their units consumed during the quarantine period were related to depression (r_s= .30, p < .0001), anxiety (r_s= .23, p= .0002), and positive urgency (r_s= .17, p= .009) (Figure 2).

[INSERT FIGURE 2 & FIGURE 2 LEGEND HERE]

DISCUSSION

We show an overall decrease in amounts and severity of problem alcohol use from pre-quarantine to the quarantine period. Critically, however, three different subpopulations were identified with most either increasing or decreasing use as compared to remaining unchanged in their alcohol use behaviours. Greater drinking was associated with demographic factors including age and male gender, COVID-19 stress-related factors, and psychiatric factors such as depression, anxiety, or the impulsivity subscale of positive urgency. Our findings underscore the theoretical mechanism of negative emotionality underlying drinking behaviours driven by stress, depression, and anxiety.

An overall decrease in alcohol use and problematic use may have multiple potential etiologies. Stringent lockdown may be associated with a decrease in the presence or availability of alcoholic beverages within the immediate household given limitations in access, a decrease in exposure to alcohol cues that may trigger urges, or the preference to consume alcohol within social contexts. More subjects reported either decreasing or increasing the frequency of their alcohol intake as compared to remaining unchanged, consistent with previous reports of a greater tendency toward extremes in individual drinking patterns when faced with either acute or chronic life stressors.[15]

Older individuals and males also showed a greater increase in drinking behaviours during lockdown and current severity of problem drinking consistent with demographic factors known to be associated with alcohol misuse. A meta-analysis focusing on gender-specific differences in

For peer review only

drinking behaviors shows that females are more likely to be lifetime non-drinkers, drink less overall, and exhibit fewer problem drinking behaviours in stressful and non-stressful contexts.[23] Also, whether one increases their drinking after experiencing acute or chronic life stress is age-dependent, which may reflect a function of previous alcohol experience.[12] Age may play a particularly unique role in the context of COVID-19 due to the greater need for stringent isolation with age, potentially fewer supports, and the risk of greater isolation, loneliness, and concern about the impact of COVID-19 on one's personal health.

COVID-19 specific stress factors appear to influence drinking behaviours controlled for other confounding variables. Being deemed an essential worker and having children was associated with a greater increase in drinking behaviours during quarantine. Importantly, although having children was associated with an increase in alcohol use, depression and anxiety scores were lower than in those without children. This suggests the additional burden of childcare and home schooling contributed to the tendency towards drinking possibly in the context of stress relief and was not mediated by greater depression or anxiety symptoms. The presence of children may also be protective against depressive and anxiety symptoms during lockdown. Having children may mitigate against loneliness that has been highlighted as a major issue during the isolation of lockdown.[24] A subset of the essential worker category – health care workers responsible for taking care of individuals with COVID-19 – was associated with greater severity of problem drinking behaviours. Thus, the specific impact of lockdown on the necessity for essential workers and the impact of the burden of home schooling and childcare on parents appears to enhance drinking behaviours independent of an impact on psychiatric symptomatology.

As expected, having a personal relationship with someone who had become severely ill or died due to COVID-19 was associated with a greater increase in severity of problem drinking behaviours. Going outdoors more frequently for work, exercise, or essential duties during lockdown was similarly associated with greater severity of alcohol use, as well as depressive and anxiety symptoms. The reasons behind the need to go outdoors complicate the interpretation, as it might be confounded by being an essential worker but also allow for greater access to the purchase of alcohol. Living with others but having a poor quality of relationship was unexpectedly associated with a lower drinking severity but with greater depressive and anxiety

For peer review only

symptoms. Living alone was not associated with any changes in drinking behaviours but was associated with greater depressive symptomatology. These findings might support the role of drinking in the context of social interactions; and further highlight the importance of social interactions during lockdown, the role of loneliness, and its impact on mental health.

We further observed a relationship between the current severity of drinking behaviours and psychiatric symptoms such as depression, anxiety or positive urgency. These relationships were driven particularly by the group which increased their drinking during quarantine. That both negative and positive emotionality factors are associated with increased drinking behaviours is in keeping with the multiple paths towards alcohol use. The effects of depression and anxiety on alcohol consumption in both AUD and non-AUD drinkers are well-documented [25-28] and related to mechanistic theories of negative emotionality, which suggest that individuals may increase their alcohol consumption in stressful contexts to cope with aversive emotional states.[3] Positive emotional factors appear to also play a role in the association with positive urgency, a subtype of impulsivity characterised by the propensity to engage in disinhibited behaviors including alcohol consumption when experiencing an intensified hedonic or excited state.[29] Positive affect-based impulsivity may reflect a heightened reward sensitivity associated with problem drinking behaviours.[30]

Limitations and future directions

This study is not without limitations. The study is a cross-sectional retrospective survey and hence potentially limited by recall bias and lack of longitudinal follow-up. Because the aim of the HabiT study was to investigate changes in frequency and severity of drinking behaviour in a large, wider population, we issued the survey internationally and during a later period of enforced isolation. Thus, the possibility cannot be overlooked that subjects were within varying phases of lockdown characterised by differential restrictions during the time of testing which may have influenced our current results. Also, approximately half of the individuals who began the survey did not complete it. This may be due to the length of the survey (i.e., 8-10 minutes). Prospective studies using an online survey design should further condense questionnaires in order to attenuate dropout. The current HabiT survey only assessed the *acute* effects of COVID-19 isolation measures on changes in drinking behaviours in comparison to the pre-quarantine

For peer review only

period. Hence, follow-up studies should be employed during the immediate post-quarantine period to investigate the possible protracted effects of COVID-19 isolation on drinking behaviours. Furthermore, whether the sampling adequately reflects the population distribution in the form of sampling bias may be an issue with online questionnaires and may under-represent those that do not have access to the internet, have limited facility with online questionnaires, or those that are more severely ill. As few respondents reported a previous history of alcohol problems relative to the expected prevalence rates, the reporting is likely either a sampling bias issue or limited willingness to reveal such a history in an online survey. This limits our capacity to assess the change in drinking behaviours in those with a history of alcohol problems. Further studies focusing specifically on the newly abstinent or those with a history of alcohol problems are indicated.

CONCLUSION

Although alcohol drinking behaviours appeared to decrease overall during lockdown, we emphasise that specific groups may be at higher risk for developing problematic alcohol use behaviours. In particular, factors associated with an increase in alcohol use include older individuals, males, essential workers, parents with children, those with a personal relationship with someone severely ill from COVID-19, and those with higher depression, anxiety levels, or positive urgency impulsivity. We emphasise that those with a previous history of alcohol misuse or a family history of AUD were not the specific focus of this study and may represent a high risk group which requires further investigation. Alcohol can be used in brief, moderate amounts in a healthy, non-pathological manner related to socialisation and stress relief. However, a subgroup of these individuals may still be at higher risk for longer term issues with alcohol misuse. The lockdown resulted in a unique set of stressors that in some cases may persist (e.g. childcare, grieving, prolonged depression or anxiety related to the lockdown) and might again re-emerge with the imposition of localised lockdowns or further lockdowns in the context of a second wave. Further studies on the longitudinal impact and persistence of these behaviours are critical. Our findings highlight a need for identifying those at greater risk for alcohol misuse to aim for greater support services and proactively target mental health issues associated with problem drinking behaviours such as depression or anxiety.

For peer review only

Funding Statement: This research was registered as a no-cost project, under grant number G107438. VV is supported by a MRC Senior Clinical Fellowship (MR/P008747/1).

Conflict of Interest Statement: All authors reported no biomedical financial interests or potential conflicts of interest.

Author Contributions: SS created the HabiT survey, collaborated with VR in analysing the collected data, and drafted and edited the manuscript. VR coded and analysed the data. HBJ collaborated with VV in conceptualising the study. VV conceptualised the study, gave crucial guidance in creating the HabiT survey, and edited the manuscript.

Data Statement: All participant data used in this research is deidentified. Participant data and MATLAB statistical code used for analysis is available upon reasonable request from corresponding author, Samantha N. Sallie, at habittstudy2020@gmail.com.

LEGENDS FOR FIGURES

Figure 1. Changes in amount and severity of drinking behaviours in the HabiT sample between pre-quarantine and quarantine periods. Units of alcohol consumed weekly (top left) and changes in drinking severity (AUDIT-C) (bottom right) decreased during the quarantine period and more individuals either increased or decreased their weekly units consumed during quarantine than remained the same (top right). Further, those who increased their weekly alcohol unit consumption during the quarantine period had significantly higher drinking severity indices (full AUDIT) compared to those who decreased or did not change their drinking behaviours during the quarantine period (bottom left).

Figure 2. Regression plots of the significant relationships between drinking severity and psychiatric measures in subjects who increased weekly alcohol unit consumption during quarantine. Drinking severity indices of the group who increased their drinking during the quarantine period were significantly positively related to depression severity, anxiety severity, and positive urgency (impulsivity subset).

For peer review only

REFERENCES

1 Holmes EA, O'Connor RC, Perry VH, et al. Multidisciplinary research priorities for the COVID-19 pandemic: a call for action for mental health science. *The Lancet Psychiatry*. 2020 Jun 1;7(6):547–60.

2 Volkow ND. Collision of the COVID-19 and addiction epidemics. *Annals of Internal Medicine*. 2020 Apr 2;173(1):61–2.

3 Koob GF. Stress, Corticotropin-releasing factor and drug addiction. *Annals of the New York Academy of Sciences*. 1999;897(1):27–45.

4 McGrath E, Jones A, Field M. Acute stress increases ad-libitum alcohol consumption in heavy drinkers, but not through impaired inhibitory control. *Psychopharmacology*. 2016 Apr 1;233(7):1227–34.

5 Marlatt GA. Taxonomy of high-risk situations for alcohol relapse: evolution and development of a. *Addiction*. 1996;91(12s1):37–50.

6 Amlung M, MacKillop J. Understanding the effects of stress and alcohol cues on motivation for alcohol via behavioral economics. *Alcoholism: Clinical and Experimental Research*. 2014;38(6):1780–9.

7 Fox HC, Bergquist KL, Hong K-I, et al. Stress-induced and alcohol cue-induced craving in recently abstinent alcohol-dependent individuals. *Alcoholism: Clinical and Experimental Research*. 2007;31(3):395–403.

8 Noone M, Dua J, Markham R. Stress, cognitive factors, and coping resources as predictors of relapse in alcoholics. *Addictive Behaviors*. 1999 Sep 1;24(5):687–93.

For peer review only

9 Cooper ML, Russell M, George WH. Coping, expectancies, and alcohol abuse: A test of social learning formulations. *Journal of Abnormal Psychology*. 1988;97(2):218–30.

10 Owens MM, Ray LA, MacKillop J. Behavioral economic analysis of stress effects on acute motivation for alcohol. *Journal of the Experimental Analysis of Behavior*. 2015;103(1):77–86.

11 Magrys SA, Olmstead MC. Acute stress increases voluntary consumption of alcohol in undergraduates. *Alcohol*. 2015 Mar 1;50(2):213–8.

12 Spanagel R, Noori HR, Heilig M. Stress and alcohol interactions: Animal studies and clinical significance. *Trends in Neurosciences*. 2014 Apr 1;37(4):219–27.

13 Clay JM, Adams C, Archer P, English M, et al. Psychosocial stress increases craving for alcohol in social drinkers: Effects of risk-taking. *Drug and Alcohol Dependence*. 2018 Apr 1;185:192–7.

14 Wit H de, Söderpalm AHV, Nikolayev L, et al. Effects of acute social stress on alcohol consumption in healthy subjects. *Alcoholism: Clinical and Experimental Research*. 2003;27(8):1270–7.

15 José BS, Van Oers H a. M, Van De Mheen HD, et al. Stressors and alcohol consumption. *Alcohol*. 2000 May 1;35(3):307–12.

16 Bush K, Kivlahan DR, McDonell MB, Fihn SD, et al. The AUDIT alcohol consumption questions (AUDIT-C): An effective brief screening test for problem drinking. *Arch Intern Med*. 1998 Sep 14;158(16):1789–95.

17 Saunders JB, Aasland OG, Babor TF, et al. Development of the alcohol use disorders identification test (AUDIT): WHO collaborative project on early detection of persons with harmful alcohol consumption-II. *Addiction*. 1993;88(6):791–804.

For peer review only

18 Snaith RP. The hospital anxiety and depression scale. *Health Qual Life Outcomes*. 2003 Aug 1;1:29.

19 Cyders MA, Littlefield AK, Coffey S, et al. Examination of a short English version of the UPPS-P impulsive behavior scale. *Addictive Behaviors*. 2014 Sep 1;39(9):1372–6.

20 MANCOVAN [Internet]. [cited 2020 Aug 13]. Available from: https://uk.mathworks.com/matlabcentral/fileexchange/27014-mancovan

21 Benjamini Y, Hochberg Y. Controlling the false discovery rate: A practical and powerful approach to multiple testing. *Journal of the Royal Statistical Society*: Series B (Methodological). 1995 Jan 1;57(1):289–300.

22 fdr_bh [Internet]. [cited 2020 Aug 13]. Available from: https://uk.mathworks.com/matlabcentral/fileexchange/27418-fdr_bh

23 Erol A, Karpyak VM. Sex and gender-related differences in alcohol use and its consequences: Contemporary knowledge and future research considerations. *Drug and Alcohol Dependence*. 2015 Nov 1;156:1–13.

24 Banerjee D, Rai M. Social isolation in Covid-19: The impact of loneliness. *Int J Soc Psychiatry*. 2020 Sep 1;66(6):525–7.

25 Peirce RS, Frone MR, Russell M, et al. A longitudinal model of social contact, social support, depression, and alcohol use. *Health Psychology*. 2000;19(1):28–38.

26 Battista S, Stewart S, Ham L. A critical review of laboratory-based studies examining the relationships of social anxiety and alcohol intake. *CDAR*. 2010 Mar 1;3(1):3–22.

27 Smith JP, Randall CL. Anxiety and alcohol use disorders. *Alcohol Res*. 2012;34(4):414–31.

For peer review only

28 Brière FN, Rohde P, Seeley JR, et al. Comorbidity between major depression and alcohol use disorder from adolescence to adulthood. *Comprehensive Psychiatry*. 2014 Apr 1;55(3):526–33.

29 Cyders MA, Smith GT. Mood-based rash action and its components: Positive and negative urgency. *Personality and Individual Differences*. 2007 Sep 1;43(4):839–50.

30 Dinc L, Cooper AJ. Positive affective states and alcohol consumption: The moderating role of trait positive urgency. *Addictive Behaviors*. 2015 Aug 1;47:17–21.

For peer review only

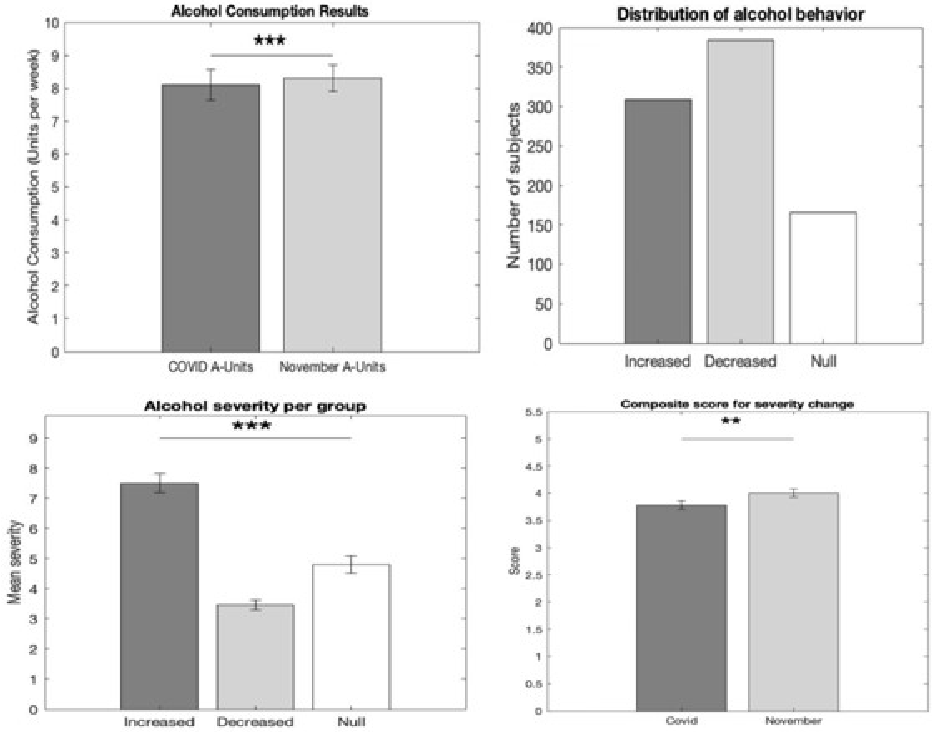

Figure 1. Changes in amount and severity of drinking behaviours in the HabiT sample between pre-quarantine and quarantine periods. Units of alcohol consumed weekly (top left) and changes in drinking severity (AUDIT-C) (bottom right) decreased during the quarantine period and more individuals either increased or decreased their weekly units consumed during quarantine than remained the same (top right). Further, those who increased their weekly alcohol unit consumption during the quarantine period had significantly higher drinking severity indices (full AUDIT) compared to those who decreased or did not change their drinking behaviours during the quarantine period (bottom left).

159x121mm (96 x 96 DPI)

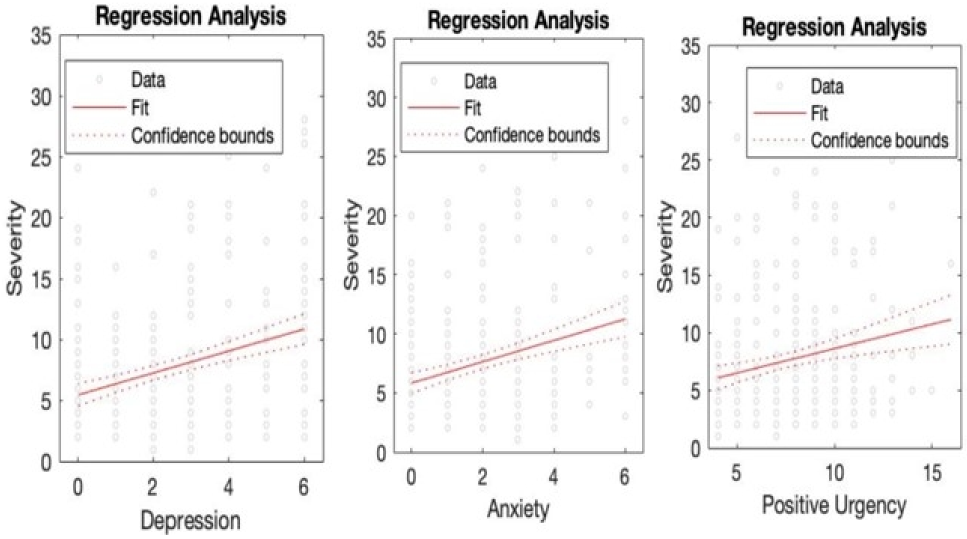

Figure 2. Regression plots of the significant relationships between drinking severity and psychiatric measures in subjects who increased weekly alcohol unit consumption during quarantine. Drinking severity indices of the group who increased their drinking during the quarantine period were significantly positively related to depression severity, anxiety severity, and positive urgency (impulsivity subset).

175x97mm (96 x 96 DPI)

Reporting checklist for cross sectional study.

Based on the STROBE cross sectional guidelines.

Instructions to authors

Complete this checklist by entering the page numbers from your manuscript where readers will find each of the items listed below.

Your article may not currently address all the items on the checklist. Please modify your text to include the missing information. If you are certain that an item does not apply, please write "n/a" and provide a short explanation.

Upload your completed checklist as an extra file when you submit to a journal.

In your methods section, say that you used the STROBE cross sectionalreporting guidelines, and cite them as:

von Elm E, Altman DG, Egger M, Pocock SJ, Gotzsche PC, Vandenbroucke JP. The Strengthening the Reporting of Observational Studies in Epidemiology (STROBE) Statement: guidelines for reporting observational studies.

For peer review only

		Reporting Item	Page Number
**Title and abstract**			
Title	#1a	Indicate the study's design with a commonly used term in the title or the abstract	1

Abstract	#1b	Provide in the abstract an informative and balanced summary of what was done and what was found	2
**Introduction**			
Background / rationale	#2	Explain the scientific background and rationale for the investigation being reported	4
Objectives	#3	State specific objectives, including any prespecified hypotheses	5
**Methods**			
Study design	#4	Present key elements of study design early in the paper	5
Setting	#5	Describe the setting, locations, and relevant dates, including periods of recruitment, exposure, follow-up, and data collection	5
Eligibility criteria	#6a	Give the eligibility criteria, and the sources and methods of selection of participants.	5
	#7	Clearly define all outcomes, exposures, predictors, potential confounders, and effect modifiers. Give diagnostic criteria, if applicable	6-7
Data sources / measurement	#8	For each variable of interest give sources of data and details of methods of assessment (measurement). Describe comparability of assessment methods if there is more than one group. Give information separately for for exposed and unexposed groups if applicable.	6-7

For peer review only

Bias	#9	Describe any efforts to address potential sources of bias	7-8
Study size	#10	Explain how the study size was arrived at	7-8
Quantitative variables	#11	Explain how quantitative variables were handled in the analyses. If applicable, describe which groupings were chosen, and why	8
Statistical methods	#12a	Describe all statistical methods, including those used to control for confounding	8
Statistical methods	#12b	Describe any methods used to examine subgroups and interactions	8
Statistical methods	#12c	Explain how missing data were addressed	7-8
Statistical methods	#12d	If applicable, describe analytical methods taking account of sampling strategy	N/A
Statistical methods	#12e	Describe any sensitivity analyses	8
**Results**			
Participants	#13a	Report numbers of individuals at each stage of study—eg numbers potentially eligible, examined for eligibility, confirmed eligible, included in the study, completing follow-up, and analysed. Give information separately for for exposed and unexposed groups if applicable.	9
Participants	#13b	Give reasons for non-participation at each stage	8

For peer review only

Participants	#13c	Consider use of a flow diagram	N/A- Cross-sectional survey design
Descriptive data	#14a	Give characteristics of study participants (eg demographic, clinical, social) and information on exposures and potential confounders. Give information separately for exposed and unexposed groups if applicable.	9
Descriptive data	#14b	Indicate number of participants with missing data for each variable of interest	9
Outcome data	#15	Report numbers of outcome events or summary measures. Give information separately for exposed and unexposed groups if applicable.	N/A- survey design
Main results	#16a	Give unadjusted estimates and, if applicable, confounder-adjusted estimates and their precision (eg, 95% confidence interval). Make clear which confounders were adjusted for and why they were included	10
Main results	#16b	Report category boundaries when continuous variables were categorized	10
Main results	#16c	If relevant, consider translating estimates of relative risk into absolute risk for a meaningful time period	N/A no risk

For peer review only

Other analyses	#17	Report other analyses done—e.g., analyses of subgroups and interactions, and sensitivity analyses	9, 12
**Discussion**			
Key results	#18	Summarise key results with reference to study objectives	13-15
Limitations	#19	Discuss limitations of the study, taking into account sources of potential bias or imprecision. Discuss both direction and magnitude of any potential bias.	15-16
Interpretation	#20	Give a cautious overall interpretation considering objectives, limitations, multiplicity of analyses, results from similar studies, and other relevant evidence.	13-15
Generalisability	#21	Discuss the generalisability (external validity) of the study results	16
**Other Information**			
Funding	#22	Give the source of funding and the role of the funders for the present study and, if applicable, for the original study on which the present article is based	16

Notes:

- 13c: N/A- Cross-sectional survey design
- 15: N/A- survey design
- 16c: N/A no risk The STROBE checklist is distributed under the terms of the Creative Commons Attribution License CC-BY. This checklist was completed on 27. August 2020 using

For peer review only

https://www.goodreports.org/, a tool made by the EQUATOR Network in collaboration with Penelope.ai

For peer review only

Assessing International Alcohol Consumption Patterns During Isolation from the COVID-19 Pandemic Using an Online Survey: Highlighting Negative Emotionality Mechanisms

Journal:	*BMJ Open*
Manuscript ID	bmjopen-2020-044276.R1
Article Type:	Original research
Date Submitted by the Author:	20-Oct-2020
Complete List of Authors:	Sallie, Samantha; University of Cambridge, Psychiatry<br>Ritou, Valentin; University of Paris, Faculty of Basic and Biomedical Sciences<br>Bowden-Jones, Henrietta; University College London, Faculty of Brain Sciences; University of Cambridge, Psychiatry<br>Voon, Valerie; Cambridge University, Psychiatry
**Primary Subject Heading**:	Mental health
Secondary Subject Heading:	Public health, Addiction
Keywords:	COVID-19, PSYCHIATRY, Substance misuse < PSYCHIATRY, PUBLIC HEALTH, Depression & mood disorders < PSYCHIATRY

SCHOLARONE™
Manuscripts

I, the Submitting Author has the right to grant and does grant on behalf of all authors of the Work (as defined in the below author licence), an exclusive licence and/or a non-exclusive licence for contributions from authors who are: i) UK Crown employees; ii) where BMJ has agreed a CC-BY licence shall apply, and/or iii) in accordance with the terms applicable for US Federal Government officers or employees acting as part of their official duties; on a worldwide, perpetual, irrevocable, royalty-free basis to BMJ Publishing Group Ltd ("BMJ") its licensees and where the relevant Journal is co-owned by BMJ to the co-owners of the Journal, to publish the Work in this journal and any other BMJ products and to exploit all rights, as set out in our licence.

The Submitting Author accepts and understands that any supply made under these terms is made by BMJ to the Submitting Author unless you are acting as an employee on behalf of your employer or a postgraduate student of an affiliated institution which is paying any applicable article publishing charge ("APC") for Open Access articles. Where the Submitting Author wishes to make the Work available on an Open Access basis (and intends to pay the relevant APC), the terms of reuse of such Open Access shall be governed by a Creative Commons licence – details of these licences and which Creative Commons licence will apply to this Work are set out in our licence referred to above.

Other than as permitted in any relevant BMJ Author's Self Archiving Policies, I confirm this Work has not been accepted for publication elsewhere, is not being considered for publication elsewhere and does not duplicate material already published. I confirm all authors consent to publication of this Work and authorise the granting of this licence.

For peer review only

Assessing International Alcohol Consumption Patterns During Isolation from the COVID-19 Pandemic Using an Online Survey: Highlighting Negative Emotionality Mechanisms

Samantha N. Sallie, MS[a]; Valentin JE. Ritou[a,b]; Henrietta Bowden-Jones, MD[a,c]; Valerie Voon, MD, PhD[a]

[a] Department of Psychiatry, University of Cambridge, Level E4, Addenbrooke's Hospital, Cambridge, United Kingdom, CB2 0QQ.

[b] Faculty of Basic and Biomedical Sciences, University of Paris (Université de Paris), 45 rue des Saints-Pères, Paris, France, 75006.

g

[c] Faculty of Brain Sciences, University College London (UCL), London, United Kingdom, WC1E 6BS.

Corresponding author: Samantha N. Sallie, MS, Department of Psychiatry, University of Cambridge, Level E4, Addenbrooke's Hospital, Cambridge, United Kingdom, CB2 0QQ, sns36@cam.ac.uk

Word Count: 4,595

For peer review only

ABSTRACT

Objectives: The Coronavirus (COVID-19) pandemic has required drastic safety measures to control virus spread, including an extended self-isolation period. Stressful situations increase alcohol craving and consumption in Alcohol Use Disorder (AUD) and non-AUD drinkers. Thus, we assessed how COVID-19-related stress may have affected drinking behaviours in the general population.

Design: We developed an online cross-sectional survey, Habit Tracker (HabiT), which measured changes in drinking behaviours before and during COVID-19 quarantine. We also assessed psychiatric factors such as anxiety, depression (HADS), and impulsivity (SUPPS-P). Lastly, we related drinking behaviours to COVID-19-specific stress factors.

Setting: HabiT was released internationally, with individuals from 83 countries participating.

Participants: Participants were included if they were 18 years of age or older, and confirmed they were proficient in English. The survey was completed by 2,873 adults with 1,346 usable data (46.9% accurately completed).

Primary Outcome Measures: Primary outcome measures were change in amount and severity of drinking behaviours before and during quarantine, and current drinking severity during quarantine.

Results: Although drinking behaviors decreased overall during quarantine, 36% reported an increase in alcohol use. Those who increased alcohol use during quarantine were older individuals(CI: 0.04-0.1, *p*<.0001), essential workers(CI: -0.58- -0.1, *p*=.01), individuals with children(CI: -12.46-0.0, *p*=.003), those with a personal relationship with someone severely ill from COVID-19(CI: -2- -0.38, *p*=.01), and those with higher depression(CI: 0.67-1.45, *p*<.0001), anxiety(CI: 0.61-1.5, *p*=.0002), and positive urgency impulsivity(CI: 0.16-0.72, *p*=.009). Further, country-level sub-sample analyses indicated that drinking amount(CI: 9.36-13.13, *p*=.003) increased in the United Kingdom during quarantine.

Conclusions: Our findings highlight a role for identifying those vulnerable for alcohol misuse during periods of self-isolation and underscore the theoretical mechanism of negative emotionality underlying drinking behaviours driven by stress. Limitations include a large degree of study dropout (n=1,515). Future studies should assess the long-term effects of isolation on drinking behaviours.

Keywords: COVID-19; alcohol use; stress; depression; self-isolation

For peer review only

ARTICLE SUMMARY

Strengths and limitations of this study

- The HabiT study sampled drinking behaviours of a large, diverse population during the COVID-19 pandemic.
- Changes in drinking behaviours were assessed against specific COVID-19-related stress factors.
- Due to the length of the survey (8-10 minutes), we observed a large degree of study dropout.
- Subjects were within varying phases of lockdown during the time of testing.
- The prevalence of diagnosed Alcohol Use Disorder drinkers sampled was low, likely related to sampling issues or under-reporting.

For peer review only

INTRODUCTION

The Coronavirus (COVID-19) pandemic has necessitated drastic safety measures to control the virus spread. These measures included an extended self-isolation period in which individuals were permitted to leave their places of residence only to obtain amenities (e.g., food, medical care, toiletries, etc.) or engage in essential work. Individuals were not permitted face-to-face contact with anyone who did not reside within their immediate households. In the United Kingdom, these measures were instituted nationally on March 23rd, 2020, with a gradual lifting of restrictions on May 10th, 2020 ending on July 4th, 2020 with locality-specific intermittent reinstatement of these measures. Although a necessary precautionary measure to mitigate the devastating effects of COVID-19 on public health, evidence indicates that protracted periods of self-isolation, especially in the context of stress, may be related to acute and prolonged negative mental health consequences, particularly in individuals already struggling with psychiatric disorders.[1]

Indeed, current clinical reports from individuals in treatment for Substance Abuse Disorder indicate that the stress produced by COVID-19 social isolation measures have triggered greater and more frequent drug or alcohol cravings, subsequently leading to relapse.[2] This observation is relevant to a prominent mechanistic theory of negative emotionality underlying alcohol misuse.[3] The relationship between stress and alcohol consumption is widely recognised and can be observed in an experimental fashion.[4] In subjects with known Alcohol Use Disorder (AUD), stress and experimental manipulations of stress enhance the amount of alcohol consumed [5, 6], alcohol craving [7], problematic drinking behaviours, and likelihood of relapse.[8] Exposure to stress triggers relapse characterised by a re-instantiation of alcohol cravings and alcohol-seeking behaviours.

Increases in alcohol craving and consumption after stress exposure also occur in those without AUD. An increase in alcohol consumption is often used as a coping strategy for both chronic and specific stressful life events in both AUD and non-AUD drinkers.[9] Similarly in both groups, self-reported craving and subjective judgements of alcohol value rise following a stress task [10], and social drinkers consume more alcohol after witnessing a social stressor.[11] These relationships are moderated by gender [12], age [13], previous alcohol exposure [13], alcohol

For peer review only - http://bmjopen.bmj.com/site/about/guidelines.xhtml

expectancies [14], and the pattern of alcohol consumption.[15] Further, psychiatric symptomology such as anxiety and depression as well as pathological levels of personality traits such as impulsivity are widely recognised predisposing factors to problematic alcohol use and addiction.[3, 16]

Thus, in response to these exceptional circumstances, we aimed to assess how social isolation measures in the midst of the COVID-19 pandemic may have affected drinking behaviours in the general adult population. We developed an international survey, entitled Habit Tracker (HabiT), which evaluated drinking severity before (post-hoc recall) and during the COVID-19 quarantine period. We hypothesised that changes in amount of alcohol consumption and severity of drinking behaviours may be related to specific COVID-19 related stress factors, as well as demographic and psychiatric factors. Further, we investigated if COVID-19-related stress factors influenced changes in drinking amount, drinking severity, depression, and anxiety before and during quarantine.

METHODS

Recruitment and inclusion criteria

The HabiT survey was a questionnaire that sought to assess the effects of isolation on alcohol, smoking, and internet use. The effects on alcohol use are reported here. Subjects were included if they were 18 years of age or older and confirmed they were proficient in reading and understanding English. HabiT was advertised by University of Cambridge news page on May 11th, 2020, a day before its international release. For the next several days, the survey was disseminated by news agencies throughout the United Kingdom (e.g., The Telegraph, BBC Cambridgeshire, News Wise) as well as throughout various University of Cambridge colleges. Further, the survey was posted and shared on personal and public social media sites (i.e., Facebook, Twitter). HabiT was approved by the Cambridge Psychology Research Ethics Committee. All subjects gave informed consent and were not financially compensated for their participation, although informed that- upon survey completion- they would be provided results of the study through request. The data collected was fully anonymised. The survey was created using Qualtrics (Provo, Utah) survey-building platform. Developed iteratively within-lab and among co-authors to insure brevity and consistency, the average time to complete the survey was

For peer review only

approximately 8-10 minutes, and all subjects could participate on either a computer or smart phone device.

Patient and public involvement statement

We did not involve patients or the public in the research design, reporting, or survet dissemination strategies of this study.

Demographic information

The demographic information collected were as follows: age, gender, socioeconomic status, intimate relationship status, country and city of residence, and any previous or current diagnosis of a psychiatric or neurological disorder.

Attentional checks

Every major section of the survey contained at least one question which served as an attentional check to ensure subjects were correctly reading and answering survey questions to the best of their ability. The attentional checks were structured to mirror the Likert scaling of each section (e.g., "If you are reading this question, please select 'Strongly Agree.'").

Frequency and severity of alcohol consumption before and during the quarantine period

We first asked subjects if they drank alcohol. If the answer was negative, they proceeded to the next set of questions. If the answer was positive, we assessed the change in the amount and severity of alcohol use as well as the current severity of alcohol use. We asked subjects to report the following behaviours within a typical week in November (i.e. pre-quarantine) and within the last week (i.e. during quarantine): (i) the number of units of alcohol consumed within the last week with examples for the number of units for differing types of alcohol and sizes provided; (ii) the change in severity using a time-scale adaptation of the first three questions of the Alcohol Use Disorders Identification Test (AUDIT-C).[17] Subjects were asked to report how many days in the last week they consumed an alcoholic beverage, how many drinks they consumed on a typical day they were drinking in the last week, and how often they consumed six or more drinks

For peer review only

on one occasion in the last week. To assess the current severity of drinking behaviours during quarantine, we used a timescale-adapted version of the full AUDIT [18] which assessed problem drinking behaviours within the last week such as an inability to stop drinking once started, failure to perform responsibilities, feeling guilt or remorse, drinking shortly after waking to ease the adverse physiological effects of drinking the night before, drinking to the point of memory loss, injuring oneself or others due to drinking, and concern from a loved one or medical professional related to the frequency or severity of one's drinking. We used two primary outcome measures: the change in severity (AUDIT-C) corroborated with the secondary change in amount of drinking (units per week) and current severity (full AUDIT).

COVID-19-related stress factors

We assessed 10 factors which may contribute to COVID-19-related stress using the following questions:

1. Have you been deemed an "essential worker" by your government?
2. Do you work for health care services specifically with individuals who have contracted Coronavirus (COVID-19)? (Sub-question of question 1)
3. Has your employment situation changed due to the Coronavirus (COVID-19) crisis?
4. Has anyone you know personally contracted or have shown symptoms characteristic of Coronavirus (COVID-19)?
5. Has anyone you know personally become severely ill or died due to contracting Coronavirus (COVID-19)?
6. Are you isolated alone?
7. Do you have children?
8. If you have children, are you their only caretaker? (Sub-question of question 7)
9. If you are currently in isolation with others, how would you describe the quality of your relations?
10. How often do you currently go outdoors (for work, essential duties, leisure, etc.)?

For peer review only

Psychiatric measures

Depression and anxiety symptomology were measured using The Hospital Anxiety and Depression Scale (HADS); a brief, validated four-item questionnaire.[19] As a secondary analysis, we assessed impulsivity using the validated Short Impulsive-Behavior Scale (SUPPS-P).[20] This scale provides an overall impulsivity score, as well as five scores corresponding to impulsivity subscales: perseveration, lack of premeditation, sensation-seeking, negative urgency, and positive urgency.

Statistical analysis

Statistical analyses were performed using MATLAB (Version 2020a). All subjects who answered the attentional checks incorrectly (n=12), reported highly improbable answers regarding the units of alcohol they consumed weekly (e.g., 1,000 units), did not report their gender, or did not complete the psychiatric questionnaires were excluded from further analysis, leaving a total of 1346 subjects. Drinking severity scores of the sample were non-normally distributed (Shapiro-Wilk, $p < .05$), thus non-parametric tests were used.

We used Mann-Whitney U-tests to compare weekly alcohol unit consumption and alcohol severity before and during quarantine in the full group. Then, we divided subjects into three groups, those who during quarantine either increased, decreased, or did not change their alcohol consumption and performed a Kruskal-Wallis H-test to assess the relative drinking amount to severity indices of these groups.

We then assessed which COVID-19-related stress factors were associated with changes in either amount (alcohol units consumed per week), change in severity (AUDIT-C), current severity (full AUDIT), or current depression and anxiety using the following tests: 1) Mann-Whitney U-Tests to compare negative versus positive responses to the COVID-19 stress factors (MW), 2) MANCOVA [21] controlling for gender and age (MAN1), and 3) A second MANCOVA controlling for age, gender, depression, and anxiety symptomology (MAN2). For the MANCOVA tests, variables ‘age,’ ‘depression severity,’ and ‘anxiety severity’ were dichotomised via median split. For the COVID-19 stress primary factor comparisons (eight items), we used False Discovery Rate (FDR) to control for multiple comparisons with

For peer review only - http://bmjopen.bmj.com/site/about/guidelines.xhtml

significance assigned at $p < .05$.[22, 23] Confidence intervals (CIs) are provided for significant findings for the most stringent statistical test.

On an exploratory basis, we then used Spearman's partial correlation to compare the drinking severity indices of subjects who completed the timescale-adapted full AUDIT with SUPPS-P and HADS scores to relate drinking severity of the overall sample to psychiatric measures. Lastly, in order to assess possible directional relationships in changes in the severity of drinking behaviors to depression, anxiety, and impulsivity; we performed Spearman's partial correlations with the psychiatric questionnaires among the three aforementioned groups (i.e., increased, decreased, and null). For both correlational analyses, we used FDR correction ($p < .05$) for multiple comparisons.

RESULTS

Demographic information

A total of 2,873 subjects participated (data collection: 05/12/2020 to 05/28/2020) of which 1,346 had usable data based on defined criteria (1,515 dropouts; 46.9% accurately completed; please refer to the supplementary materials for a demographic analysis of those who did not complete the survey). Of these subjects, 859 (63.8%) reported that they drink alcohol (please refer to the supplementary materials for demographic information for those report drinking alcohol). Of the 1346 subjects, the average age was 28.92 ± 10.45 years [CI: 28.2-29.53] (range= 18-90) with more males (males: n= 1006; females: n=325; other: n=15) from 85 different countries of residence, with the majority from the United Kingdom (n= 434) and the United States (n= 355), followed by Canada (n= 64) and Germany (n= 63). Marital status was as follows: single: n=785; married or committed: n=571; divorced or separated: n=33; widowed: n=4. Socioeconomic status (as denoted by annual income) was as follows: <19.9k: n=285; 20-39.9k: n= 273; 20-39.9k: n=244; 40-69.9k: n=241; 70-99.9k: n=141; >100k: n=203; and 232 subjects did not report their incomes. Current psychiatric or neurological diagnoses were as follows: no diagnosis: n=1192; depression: n= 60; anxiety: n= 38, Post-Traumatic Stress Disorder (PTSD): n= 5, comorbid depression and anxiety: n= 46.

For peer review only - http://bmjopen.bmj.com/site/about/guidelines.xhtml

Overall changes in drinking frequency and severity before and during quarantine

Of the total sample, the change in problem drinking severity (AUDIT-C) was in 0.89 ± 1.43 [CI: 0.81-0.96] (range: 0-8) and the mean change in the amount consumed was 5.62 ± 9.55 units per week [CI: 3.16-4.02] (range: 0-120). The current problem drinking severity (full AUDIT) was 3.14 ± 4.47 [CI: 2.9-3.37] (range: 0-32), with 557 subjects included that do not consume alcohol. Of the subjects who reported they consume alcohol (n= 859), the change in severity from pre-quarantine to quarantine was a decrease of 1.53 ± 1.6, [CI: 5.01-5.64] range 0-8 (U= 2.65, [CI: 0-0.21] *p*= .008). The units of alcohol consumed per week was significantly decreased during the quarantine period (8.03 ± 14.22 units, [7.11-8.94] range= 1-120) compared to November (8.32 ± 11.92 units, [CI: 7.47-9.02] range = 0-150), U= -2.29, [CI: 0.0-0.0] *p*= .02 (Figure 1). However, in the UK, the units of alcohol consumed per week was significantly increased during the quarantine period (11.25 ± 17.73 units, [CI: 9.36-13.13] range= 1-120) compared to November (10.94 ± 14.17 units, [CI: 9.44-12.45] range = 0-150), U= 3.0, [CI: 0-0.7] *p*= .003. (For full country-level sub-analyses of change in weekly drinking amount, change in severity, and overall severity during quarantine, please refer to the supplementary materials). Of the international sample, 172 (20%) subjects reported abstention from alcohol consumption during the quarantine period.. More subjects reported a decrease (n= 384, 45%) or an increase (n= 308, 36%) as opposed to no change (n= 166, 19%) of weekly alcohol consumption from November to the quarantine period (X^2= 72.86, *p*= .001; Figure 1).. Of the three groups, those who: 1) increased weekly units consumed during quarantine (7.5 ± 10.5 change in units, [CI: 6.33-8.7] range: 1-80), 2) decreased weekly units consumed during quarantine (-6.5 ± 9.5 change in units, [CI: -7.45- -5.55] range: -.2 - -120), and 3) did not change their weekly unit consumption, subjects who had increased the units of alcohol consumed during the quarantine period showed significantly higher current drinking severity scores (7.5 ± 5.6, [CI: 6.89-8.15] range: 1-32) than those who reported decreases (3.5 ± 3.0, [CI: 3.16-3.76] range: 1-21) or no changes (4.8 ± 3.6, [CI: 4.17-5.23] range: 1-20) in weekly unit consumption (H= 165.33, [CI: 3.35-4.78] *p* < .0001, Figure 1).

[INSERT FIGURE 1 & FIGURE 1 LEGEND HERE]

For peer review only

COVID-19 stress factor evaluation

The change in amount of drinking was positively correlated with age ($r_s = 0.2$, [CI: 0.04-0.1] $p < .0001$), and gender with males (6.44 ± 10.8 units, [CI: 5.63-7.35] range: 0-120) showing an increased change in drinking amount relative to females (3.81 ± 5.18, [CI: 3.08-4.32] range: 0-38) or other genders (1.32 ± 1.65, [CI: 0.18-2.24] range: 0-5) (H= 8.17, $p = .003$). Changes in drinking severity were also related to both age and gender, with older individuals ($r_s = .2$, [CI: 0.01-0.02] $p < .0001$) and males (1.68 ± 1.74, [CI: 1.55-1.83] range: 0-8) demonstrating greater changes in their drinking severity than females (1.16 ± 1.12, [CI: 1.02-1.3] range: 0-8) and others (1.36 ± 1.29, [CI: 0.54-2.18] range: 0-3) (H= 6.02, [CI: -0.81- -0.22] $p = .05$). (Gender-specific sub-analyses of drinking behaviours can be found in the supplementary materials). Thus, we utilised age and gender as covariates for both MANCOVA analyses. All relevant covariates used in these analyses were dichotomised via median split (age= 25.1 years, depression severity= 2, and anxiety severity= 1).

Primary COVID-19 stress factors

The influence of COVID-19 stress factors on the change in drinking severity, amounts consumed, and current drinking severity are reported in Tables 1, 2, and 3, respectively. Designated essential workers and those with children showed a greater increase in the amount consumed weekly and drinking severity as well as greater current severity. This remained significant including when controlled for demographic variables (age, gender) and psychiatric symptoms (depression, anxiety). Notably, although subjects with children reported an increase in the number of units of alcohol and severity of alcohol use, they also reported lower levels of depression and anxiety. Knowing an individual personally who was ill or severely ill with Covid-19 showed higher current alcohol drinking severity than those who did not, but with no change from pre- to post-quarantine. A reported change in employment status and isolating alone was associated with greater depression scores, with no differences in drinking behaviours. Isolating with others but reporting a poor relationship was associated with greater depression and anxiety, however, the lower drinking behaviours were moderated by age and gender effects. Finally, going outdoors was associated with greater current drinking severity and greater depression and anxiety scores controlling for all variables. Post-hoc tests confirmed that, in cases in which a significant relationship was lost between an item and either changes in drinking frequency or

severity due to controlling for age and gender (i.e., MANCOVA 1), age was the sole contributor (Essential worker: F(1, 533.2)= 7, [CI: 0.15-2.1] *p*= .008; Others ill: F(1, 879.9)= 52.6, [CI: 1.7-2.7] *p* < .0001; Poor relationship: F(1, 933.9)= 48.88, [CI: 1.8-2.8] *p* < .0001).

Stress Factor	N Total	Yes M(SD)	N Yes	No M(SD)	N No	MW p-value	MAN1 p-value	MAN2 p-value	CI
Essential worker	1337	0.16(1.9)	241	-0.21(1.6)	1096	0.02*	0.01*	0.01*	-0.58- -0.1
Employment	1337	-0.14(1.8)	323	-0.14(1.6)	1014	0.83	0.96	0.92	
Others ill	1334	-0.17(1.8)	497	-0.12(1.6)	837	0.75	0.64	0.63	
Others severely ill	1336	-0.01(2)	127	-0.15(1.6)	1209	0.35	0.7	0.69	
Isolated alone	1325	-0.1(1.9)	168	-0.15(1.6)	1157	0.83	0.85	0.82	
Having children	1334	0.34(1.4)	209	-0.23(1.7)	1125	<.0001*	0.005*	0.003*	-12.46-0.0
Poor relationship	1168	-0.3(1.7)	187	-0.13(1.6)	981	0.35	0.7	0.69	
Going outdoors	1336	-0.27(1.3)	193	-0.12(1.7)	1143	0.26	0.7	0.69	

Table 1. COVID-19 primary stress items relationship with changes in drinking severity (as indexed by the AUDIT-C) from pre-quarantine to quarantine.

Stress Factor	N Total	Yes M(SD)	N Yes	No M(SD)	N No	MW p-value	MAN1 p-value	MAN2 p-value	CI
Essential worker	1337	1.26(12.8)	241	0.45(7.5)	1096	0.0003*	0.07	0.08	-3.4- -0.02
Employment	1337	0.17(11.2)	323	0.13(7.8)	1014	0.77	0.95	0.97	
Others ill	1334	0.05(7.1)	497	0.2(9.6)	837	0.83	0.95	0.97	
Others severely ill	1336	0.06(7.6)	127	0.15(8.9)	1209	0.83	0.95	0.97	
Isolated alone	1325	0.05(11.6)	168	0.2(8.2)	1157	0.46	0.95	0.97	
Having children	1334	2.02(11.9)	209	0.54(7.9)	1125	<.0001*	0.04*	0.02*	-3.6- - 0.74
Poor relationship	1168	0.4(6.1)	187	0.19(8.7)	981	0.46	0.95	0.97	
Going outdoors	1336	1.23(6.8)	193	0.04(9.0)	1143	0.15	0.47	0.4	

Table 2. COVID-19 primary stress items relationship with changes in drinking amount (in units) from pre-quarantine to quarantine.

Stress Factor	N Total	Severity Type	Yes M(SD)	N Yes	N M(SD)	N No	M-W p-value	MAN1 p-value	MAN2 p-value	CI
Essential worker	1337	Drinking	4.42(5.7)	243	2.85(4.1)	1099	<.0001*	0.0005*	0.0005*	-1.8- -057
		Depression	2.29(1.8)	243	2.44(1.9)	1099	0.43	0.84		
		Anxiety	1.79(1.7)	243	1.94(1.8)	1099	0.42	0.8		
Employment change	1337	Drinking	3.46(4.9)	324	3.02(4.3)	1018	0.38	0.08	0.144	
		Depression	2.78(2.0)	324	2.31(1.9)	1018	0.0043*	0.007*		-0.58- -0.1
		Anxiety	2.03(4.5)	324	1.88(1.8)	1018	0.32	0.363		
Others ill	1334	Drinking	3.59(1.9)	499	2.87(4.4)	840	<.0001*	0.1	0.125	-1.2- -0.2
		Depression	2.3(1.8)	499	2.47(1.9)	840	0.20	0.83		
		Anxiety	1.9(5.5)	499	1.93(1.9)	840	0.99	0.94		
Others severely ill	1336	Drinking	4.49(2.0)	127	2.99(4.3)	1214	0.001*	0.007*	0.01*	-2- -0.38
		Depression	2.45(2.0)	127	2.4(1.9)	1214	0.99	0.41		
		Anxiety	1.92(5.8)	127	1.91(1.8)	1214	0.82	0.84		
Isolated alone	1325	Drinking	3.88(2.0)	169	2.98(4.2)	1161	0.42	0.83	0.87	
		Depression	3.4(1.9)	169	2.41(1.9)	1161	0.009*	0.04*		-0.7- -0.06
		Anxiety	2.04(5.2)	169	1.9(1.8)	1161	0.43	0.11		

Having children	1334	Drinking	5.17(1.8)	211	2.75(4.2)	1128	< 0001*	0.0003*	<.0001*	-2.4- -0.9
		Depression	1.5(1.7)	211	2.58(1.9)	1128	<.0001*	<.0001*		0.37-0.97
		Anxiety	1.37(1.7)	211	2.02(1.9)	1128	<.0001*	0.0009*		0.25-0.85
Poor relationship	1168	Drinking	2.82(5.1)	187	3.1(4.1)	985	0.01*	0.92	0.87	0.4- 1.0
		Depression	3.57(2.0)	187	2.2(1.8)	985	<.0001*	<.0001*		-1.53- -1
		Anxiety	2.79(2.0)	187	1.74(1.8)	985	<.0001*	<.0001*		-1.3- -073
Going outdoors	1336	Drinking	3.42(4.5)	1148	1.37(3.4)	193	<.0001*	<.0001*	<.0001*	1.14-2.47
		Depression	3.18(2.0)	193	2.28(1.9)	1148	<.0001*	<.0001*		-1- -0.42
		Anxiety	2.42(2.0)	193	1.83(1.8)	1148	0.0002*	0.0008*		-0.8- -0.24

Table 3. COVID-19 primary stress items relationship with current drinking severity (i.e., full AUDIT), depression, and anxiety from pre-quarantine to quarantine.

Secondary COVID-19 stress factors

Two COVID-19 stress factors were considered secondary as they represented a subset of a primary factor. Working for health care services was associated with a trend towards a greater change in amount of units consumed (F= 3.97 [CI: -6.73- -0.0], *p* = .05) and greater severity of current drinking (F= 7.01, [CI: -3.9- -0.6] *p* = .007) when controlled for all variables. Being the only caretaker for children was also associated with greater change in drinking severity (U= 2.62, [CI: -2.7- -0.9] *p* = .009) and greater change of amount consumed (U= 2.67, [CI: -4.5- -0.8] *p* = .007), but was no longer significant when controlling for age and gender.

Drinking severity during quarantine and correlations with psychiatric measures

Of the individuals who reported drinking alcohol, (n= 769) completed the current drinking severity index (e.g., the adapted-timescale full AUDIT). The severity of drinking behaviours was positively related to depression (r_s= .12, [CI: 0.34-.79] *p*= .004), anxiety (r_s= .12, [CI: 0.3-0.74] *p*= .027), and positive urgency impulsivity (r_s= .12, [CI: 0.14-0.34] *p*= .004), controlled for age and gender. To assess potential directional relationships between current drinking severity during quarantine and psychiatric measures, we correlated depression, anxiety, and impulsivity with the three drinking groups (i.e., increased, decreased, null). Drinking severity scores in the decreased and no change groups were not significantly correlated with any of the psychiatric measures of interest. However, drinking severity of those who increased their units consumed during the quarantine period were related to depression (r_s= .30, [CI: 0.67-1.45] *p* < .0001), anxiety (r_s= .23, [CI: 0.61-1.5] *p*= .0002), and positive urgency (r_s= .17, [CI: 0.16-0.72] *p*= .009) (Figure 2).

[INSERT FIGURE 2 & FIGURE 2 LEGEND HERE]

DISCUSSION

We show an overall decrease in amounts and severity of problem alcohol use from pre-quarantine to the quarantine period. Critically, however, three different subpopulations were identified with most either increasing or decreasing use as compared to remaining unchanged in their alcohol use behaviours. Greater drinking was associated with demographic factors including age and male gender, COVID-19 stress-related factors, and psychiatric factors such as depression, anxiety, or the impulsivity subscale of positive urgency. Our findings underscore the theoretical mechanism of negative emotionality underlying drinking behaviours driven by stress, depression, and anxiety.

An overall decrease in alcohol use and problematic use may have multiple potential etiologies. Stringent lockdown may be associated with a decrease in the presence or availability of alcoholic beverages within the immediate household given limitations in access, a decrease in exposure to alcohol cues that may trigger urges, or the preference to consume alcohol within social contexts. More subjects reported either decreasing or increasing the frequency of their alcohol intake as compared to remaining unchanged, consistent with previous reports of a greater tendency toward extremes in individual drinking patterns when faced with either acute or chronic life stressors.[15]

Older individuals showed a greater increase in drinking behaviours during lockdown and current severity of problem drinking consistent with demographic factors known to be associated with alcohol misuse. Whether one increases their drinking after experiencing acute or chronic life stress is age-dependent, which may reflect a function of previous alcohol experience.[13] Age may play a particularly unique role in the context of COVID-19 due to the greater need for stringent isolation with age, potentially fewer supports, and the risk of greater isolation, loneliness, and concern about the impact of COVID-19 on one's personal health. Expectedly, males showed greater unit consumption compared to females and other genders overall. However, males showed a decrease in both drinking amount and severity during quarantine,

For peer review only

while females demonstrated the opposite trend. This finding corroborates evidence which indicates females are more likely than males to consume alcohol in order to cope with stress.[24]

COVID-19 specific stress factors appear to influence drinking behaviours controlled for other confounding variables. Being deemed an essential worker and having children was associated with a greater increase in drinking behaviours during quarantine. Importantly, although having children was associated with an increase in alcohol use, depression and anxiety scores were lower than in those without children. This suggests the additional burden of childcare and home schooling contributed to the tendency towards drinking possibly in the context of stress relief and was not mediated by greater depression or anxiety symptoms. The presence of children may also be protective against depressive and anxiety symptoms during lockdown. Having children may mitigate against loneliness that has been highlighted as a major issue during the isolation of lockdown.[25] A subset of the essential worker category – health care workers responsible for taking care of individuals with COVID-19 – was associated with greater severity of problem drinking behaviours. Thus, the specific impact of lockdown on the necessity for essential workers and the impact of the burden of home schooling and childcare on parents appears to enhance drinking behaviours independent of an impact on psychiatric symptomatology.

As expected, having a personal relationship with someone who had become severely ill or died due to COVID-19 was associated with a greater increase in severity of problem drinking behaviours. Going outdoors more frequently for work, exercise, or essential duties during lockdown was similarly associated with greater severity of alcohol use, as well as depressive and anxiety symptoms. The reasons behind the need to go outdoors complicate the interpretation, as it might be confounded by being an essential worker but also allow for greater access to the purchase of alcohol. Living with others but having a poor quality of relationship was unexpectedly associated with a lower drinking severity but with greater depressive and anxiety symptoms. Living alone was not associated with any changes in drinking behaviours but was associated with greater depressive symptomatology. These findings might support the role of drinking in the context of social interactions; and further highlight the importance of social interactions during lockdown, the role of loneliness, and its impact on mental health.[25]

For peer review only

Importantly, those residing in the UK- unlike those in the US and Canada- displayed an increase in weekly alcohol units consumed during quarantine, consistent with the WHO Global Status Report on Alcohol and Health (2018) which shows that total alcohol per capita consumption (APC) is higher in the UK than in the US or Canada.[26]

We further observed a relationship between the current severity of drinking behaviours and psychiatric symptoms such as depression, anxiety or positive urgency. These relationships were driven particularly by the group which increased their drinking during quarantine. That both negative and positive emotionality factors are associated with increased drinking behaviours is in keeping with the multiple paths towards alcohol use. The effects of depression and anxiety on alcohol consumption in both AUD and non-AUD drinkers are well-documented [27-30] and related to mechanistic theories of negative emotionality, which suggest that individuals may increase their alcohol consumption in stressful contexts to cope with aversive emotional states.[31] Positive emotional factors appear to also play a role in the association with positive urgency, a subtype of impulsivity characterised by the propensity to engage in disinhibited behaviors including alcohol consumption when experiencing an intensified hedonic or excited state.[30] Positive affect-based impulsivity may reflect a heightened reward sensitivity associated with problem drinking behaviours.[32]

Limitations and future directions

This study is not without limitations. HabiT is a cross-sectional, retrospective survey and hence potentially limited by recall and misclassification biases as well as lack of longitudinal follow-up. Because retrospective reporting involves issues with memory, possible Dunning-Kruger effects, and selection bias; the reader should be cautious in drawing causal interpretations from the current data. Because the aim of the HabiT study was to investigate changes in frequency and severity of drinking behaviour in a large, wider population, we issued the survey internationally and during a later period of enforced isolation. Thus, the possibility cannot be overlooked that subjects were within varying phases of lockdown characterised by differential restrictions during the time of testing which may have influenced our current results. Future studies may consider data analysis by country, level of lockdown, or amount and severity of localised COVID-19

For peer review only

cases. Also, approximately half of the individuals who began the survey did not complete it. This may be due to the length of the survey (i.e., 8-10 minutes). Prospective studies using an online survey design should further condense questionnaires and/or offer subjects monetary incentives obtained upon survey completion in order to attenuate dropout and non-response bias. The current HabiT survey only assessed the *acute* effects of COVID-19 isolation measures on changes in drinking behaviours in comparison to the pre-quarantine period. Hence, follow-up studies should be employed during the immediate post-quarantine period to investigate the possible protracted effects of COVID-19 isolation on drinking behaviours. Furthermore, whether the sampling adequately reflects the population distribution in the form of sampling bias may be an issue with online questionnaires and may under-represent those who do not have smartphones or access to the internet [33], have limited facility with online questionnaires (e.g., older individuals) [33], were otherwise engaged (e.g., caring for an ill individual or children), or are more severely ill with substance use or other mental health disorders. Thus, our ability to generalise our current findings to the wider population is limited. Other methods (e.g., phone surveys) are recommended to reach populations under-represented by online surveys.[34] As few respondents reported a previous history of alcohol problems relative to the expected prevalence rates, the reporting is likely either a function of sampling bias, limited willingness to reveal such a history in an online survey, or marked changes in alcohol use particularly if relapse occurs. This limits our capacity to assess the change in drinking behaviours in those with a history of alcohol problems. Further studies focusing specifically on the newly abstinent or those with a history of alcohol problems are indicated.

CONCLUSION

Although alcohol drinking behaviours appeared to decrease overall during lockdown, we emphasise that specific groups may be at higher risk for developing problematic alcohol use behaviours. In particular, factors associated with an increase in alcohol use include older individuals, essential workers, parents with children, those with a personal relationship with someone severely ill from COVID-19, and those with higher depression, anxiety levels, or positive urgency impulsivity. Further, unlike residents from the US and Canada, those in the UK increased their weekly alcohol intake during the quarantine period. We emphasise that those with

For peer review only

a previous history of alcohol misuse or a family history of AUD were not the specific focus of this study and may represent a high risk group which requires further investigation. Alcohol can be used in brief, moderate amounts in a healthy, non-pathological manner related to socialisation and stress relief. However, a subgroup of these individuals may still be at higher risk for longer term issues with alcohol misuse. The lockdown resulted in a unique set of stressors that in some cases may persist (e.g. childcare, grieving, prolonged depression or anxiety related to the lockdown) and might again re-emerge with the imposition of localised lockdowns or further lockdowns in the context of a second wave. Further studies on the longitudinal impact and persistence of these behaviours are critical. Our findings highlight a need for identifying those at greater risk for alcohol misuse to aim for greater support services and proactively target mental health issues associated with problem drinking behaviours such as depression or anxiety.

Funding Statement: This research was registered as a no-cost project, under grant number G107438. VV is supported by a MRC Senior Clinical Fellowship (MR/P008747/1).

Conflict of Interest Statement: All authors reported no biomedical financial interests or potential conflicts of interest.

Author Contributions: SS created the HabiT survey, collaborated with VR in analysing the collected data, and drafted and edited the manuscript. VR coded and analysed the data. HBJ collaborated with VV in conceptualising the study. VV conceptualised the study, gave crucial guidance in creating the HabiT survey, and edited the manuscript.

Data Statement: All participant data used in this research is deidentified. Participant data and MATLAB statistical code used for analysis is available upon reasonable request from corresponding author, Samantha N. Sallie, at habittstudy2020@gmail.com.

For peer review only

LEGENDS FOR FIGURES

Figure 1. Changes in amount and severity of drinking behaviours in the HabiT sample between pre-quarantine and quarantine periods. Units of alcohol consumed weekly (top left) and changes in drinking severity (AUDIT-C) (bottom right) decreased during the quarantine period and more individuals either increased or decreased their weekly units consumed during quarantine than remained the same (top right). Further, those who increased their weekly alcohol unit consumption during the quarantine period had significantly higher drinking severity indices (full AUDIT) compared to those who decreased or did not change their drinking behaviours during the quarantine period (bottom left).

Figure 2. Regression plots of the significant relationships between drinking severity and psychiatric measures in subjects who increased weekly alcohol unit consumption during quarantine. Drinking severity indices of the group who increased their drinking during the quarantine period were significantly positively related to depression severity, anxiety severity, and positive urgency (impulsivity subset).

For peer review only

REFERENCES

1 Holmes EA, O'Connor RC, Perry VH, et al. Multidisciplinary research priorities for the COVID-19 pandemic: a call for action for mental health science. *The Lancet Psychiatry*. 2020 Jun 1;7(6):547–60.

2 Volkow ND. Collision of the COVID-19 and addiction epidemics. *Annals of Internal Medicine*. 2020 Apr 2;173(1):61–2.

3 Koob GF. Stress, Corticotropin-releasing factor and drug addiction. *Annals of the New York Academy of Sciences*. 1999;897(1):27–45.

4 McGrath E, Jones A, Field M. Acute stress increases ad-libitum alcohol consumption in heavy drinkers, but not through impaired inhibitory control. *Psychopharmacology*. 2016 Apr 1;233(7):1227–34.

5 Marlatt GA. Taxonomy of high-risk situations for alcohol relapse: evolution and development of a. *Addiction*. 1996;91(12s1):37–50.

6 Amlung M, MacKillop J. Understanding the effects of stress and alcohol cues on motivation for alcohol via behavioral economics. *Alcoholism: Clinical and Experimental Research*. 2014;38(6):1780–9.

7 Fox HC, Bergquist KL, Hong K-I, et al. Stress-induced and alcohol cue-induced craving in recently abstinent alcohol-dependent individuals. *Alcoholism: Clinical and Experimental Research*. 2007;31(3):395–403.

8 Noone M, Dua J, Markham R. Stress, cognitive factors, and coping resources as predictors of relapse in alcoholics. *Addictive Behaviors*. 1999 Sep 1;24(5):687–93.

9 Cooper ML, Russell M, George WH. Coping, expectancies, and alcohol abuse: A test of social learning formulations. *Journal of Abnormal Psychology*. 1988;97(2):218–30.

For peer review only

10 Owens MM, Ray LA, MacKillop J. Behavioral economic analysis of stress effects on acute motivation for alcohol. *Journal of the Experimental Analysis of Behavior*. 2015;103(1):77–86.

11 Magrys SA, Olmstead MC. Acute stress increases voluntary consumption of alcohol in undergraduates. *Alcohol*. 2015 Mar 1;50(2):213–8.

12 Wilsnack RW, Wilsnack SC, Kristjanson AF, Vogeltanz-Holm ND, Gmel G. Gender and alcohol consumption: Patterns from the multinational genacis project. *Addiction*. 2009 Sep;104(9):1487–500.

13 Spanagel R, Noori HR, Heilig M. Stress and alcohol interactions: Animal studies and clinical significance. *Trends in Neurosciences*. 2014 Apr 1;37(4):219–27.

14 Clay JM, Adams C, Archer P, English M, et al. Psychosocial stress increases craving for alcohol in social drinkers: Effects of risk-taking. *Drug and Alcohol Dependence*. 2018 Apr 1;185:192–7.

1.

15 José BS, Van Oers H a. M, Van De Mheen HD, Garretsen HFL, Mackenbach JP. Stressors and alcohol consumption. *Alcohol*. 2000 May 1;35(3):307–12.

16 Dick DM, Smith G, Olausson P, Mitchell SH, Leeman RF, O'Malley SS, et al. Understanding the construct of impulsivity and its relationship to alcohol use disorders. Addict Biol. 2010 Apr;15(2):217–26.

17 Bush K, Kivlahan DR, McDonell MB, Fihn SD, et al. The AUDIT alcohol consumption questions (AUDIT-C): An effective brief screening test for problem drinking. *Arch Intern Med*. 1998 Sep 14;158(16):1789–95.

For peer review only

18 Saunders JB, Aasland OG, Babor TF, et al. Development of the alcohol use disorders identification test (AUDIT): WHO collaborative project on early detection of persons with harmful alcohol consumption-II. *Addiction*. 1993;88(6):791–804.

19 Snaith RP. The hospital anxiety and depression scale. *Health Qual Life Outcomes*. 2003 Aug 1;1:29.

20 Cyders MA, Littlefield AK, Coffey S, et al. Examination of a short English version of the UPPS-P impulsive behavior scale. *Addictive Behaviors*. 2014 Sep 1;39(9):1372–6.

21 MANCOVAN [Internet]. [cited 2020 Aug 13]. Available from: https://uk.mathworks.com/matlabcentral/fileexchange/27014-mancovan

22 Benjamini Y, Hochberg Y. Controlling the false discovery rate: A practical and powerful approach to multiple testing. *Journal of the Royal Statistical Society*: Series B (Methodological). 1995 Jan 1;57(1):289–300.

23 fdr_bh [Internet]. [cited 2020 Aug 13]. Available from: https://uk.mathworks.com/matlabcentral/fileexchange/27418-fdr_bh

24 Peltier MR, Verplaetse TL, Mineur YS, Petrakis IL, Cosgrove KP, Picciotto MR, et al. Sex differences in stress-related alcohol use. Neurobiology of Stress. 2019 Feb 1;10:100149.

25 Banerjee D, Rai M. Social isolation in Covid-19: The impact of loneliness. *Int J Soc Psychiatry*. 2020 Sep 1;66(6):525–7.

26 Global status report on alcohol and health 2018. Geneva: World Health Organization; 2018. Licence: CC BY-NC-SA 3.0 IGO

27 Peirce RS, Frone MR, Russell M, et al. A longitudinal model of social contact, social support, depression, and alcohol use. *Health Psychology*. 2000;19(1):28–38.

For peer review only

28 Battista S, Stewart S, Ham L. A critical review of laboratory-based studies examining the relationships of social anxiety and alcohol intake. *CDAR*. 2010 Mar 1;3(1):3–22.

29 Smith JP, Randall CL. Anxiety and alcohol use disorders. *Alcohol Res*. 2012;34(4):414–31.

30 Brière FN, Rohde P, Seeley JR, et al. Comorbidity between major depression and alcohol use disorder from adolescence to adulthood. *Comprehensive Psychiatry*. 2014 Apr 1;55(3):526–33.

31 Cyders MA, Smith GT. Mood-based rash action and its components: Positive and negative urgency. *Personality and Individual Differences*. 2007 Sep 1;43(4):839–50.

32 Dinc L, Cooper AJ. Positive affective states and alcohol consumption: The moderating role of trait positive urgency. *Addictive Behaviors*. 2015 Aug 1;47:17–21.

33 Remillard ML, Mazor KM, Cutrona SL, et al. Systematic review of the use of online questionnaires among the geriatric population. *J Am Geriatr Soc*. 2014 Apr;62(4):696–705.

34 Szolnoki G, Hoffmann D. Online, face-to-face and telephone surveys—Comparing different sampling methods in wine consumer research. *Wine Economics and Policy*. 2013 Dec 1;2(2):57–66.

For peer review only

SUPPLEMENTARY MATERIALS

Demographics for drinkers

	Age		Sex		Country		SES		Relationship
Mean	31.4	Male	599	Total	49	Lower	328	Single	449
SD	13.2	Female	248	UK	347	Mid	176	Relationship	408
Range	18-90	Other	12	US	223	Higher	250		

Depression	Anxiety	PTSD	Depression & Anxiety
41	27	3	35

For peer review only

Demographic analysis for study dropouts

Although a majority of the dropout subjects (n=1,515) who entered the study provided no data (n=981), we performed a demographic analysis on dropout subjects who provided this information (n=481) to assess if those who completed the survey differed in demographic factors from those who did not. The mean age of dropout subjects was 26.58 ± 11.11 years [CI: 25.59-27.58] (range= 18-80 years), significantly younger than the mean of age of individuals who completed the survey (U= 3.69, [CI: 1.15-3.54] $p < .0001$). Further, more males (n=387) than females (n=87) or other genders (n=7) dropped out of the study prior to completion ($X^2= 61.23$, $p < .0001$).

Sub-sample analysis by country

United Kingdom (UK)

In the UK, the change in problem drinking severity (AUDIT-C) was 1.05 ± 1.46 [CI: 0.91-1.19] (range: 0-8), and the mean change in the amount consumed was 5.93 ± 11.75 [CI: 4.82-7.05], units per week (range: 0-120). Current problem drinking severity (full AUDIT) was 4.09 ± 4.94 [CI: 3.62-4.56] (range: 0-27). Of the subjects who reported they consume alcohol (n=434), the change in severity from pre-quarantine to quarantine was a decrease of -0.16 ± 2.15, [CI: -0.3-0.06] (range -8-6) but not significantly so (U= -1.38, [CI: 0.01-0.89] $p = .19$). The units of

alcohol consumed per week was significantly increased during the quarantine period (11.25 ± 17.73 units, [CI: 9.36-13.13] range= 1-120) compared to November (10.94 ± 14.17 units, [CI: 9.44-12.45] range = 0-150), U= 3.0, [CI: 0-0.7] p= .003. Further, 60 (14%) subjects reported abstention from alcohol consumption during the quarantine period. More subjects reported a decrease (n= 151, 43%) or an increase (n= 130, 39%) as opposed to no change (n= 61, 18%) of weekly alcohol consumption from November to the quarantine period (X^2= 7.2, p = .007).

United States (US)

In the US, change in problem drinking severity (AUDIT-C) was 1.01 ± 1.55 units [CI: 0.85-1.17] (range: 0-8), and the mean change in the amount consumed was 3 ± 5.51 [CI: 2.39-4] units per week (range: 0-34). The current problem drinking severity (full AUDIT) was 3.48 ± 4.95 [CI: 3-4] (range: 0-32). Of the subjects who reported they consume alcohol (n= 353), the change in severity from pre-quarantine to quarantine was a decrease of -0.11 ± 2.42 [CI: -0.43-0.21], range -8-8 (U= -0.66, [CI: 0.05-0.9] p= .51), but not significantly so. The units of alcohol consumed per week increased between the quarantine period (7.39 ± 11.45 units, [CI: 5.88-8.9] range= 0-80) and November (6.93 ± 9.78 units, [CI: 5.88-8.9] range = 0-96), but not significantly so (U= -1.1, [CI: 0.01-0.94] p= .29). Further, 44 (13%) subjects reported abstention from alcohol consumption during the quarantine period. More subjects reported a decrease (n= 90, 41%) or an increase (n= 88, 40%) as opposed to no change (n= 45, 21%) of weekly alcohol consumption from November to the quarantine period (X^2= 8.15, p = .004).

Canada

In Canada, change in problem drinking severity (AUDIT-C) was 0.67 ± 1.45 [CI: 0.31-1.03] (range: 0-8), and the mean change in the amount consumed was 3.03 ± 7.45 [CI: 1.17-4.89] units per week (range: 0-49). The current problem drinking severity (full AUDIT) was 2.78 ± 4.24 [CI: 1.7-3.85] (range: 0-24). Of the subjects who reported they consume alcohol (n= 35), the change in severity from pre-quarantine to quarantine was an increase of 0.16 ± 2.2, [CI: -0.62-0.95](range= -8-5), but not significantly so (U= .77, [CI: 0.03-0.98] p= .44). The units of alcohol consumed per week was decreased during the quarantine period (8.03 ± 14.22 units, [CI:] range= 0-50) and November (6.71 ± 9.49 units, [CI: 3.46-9.97] range = 0-25), although not

For peer review only

significantly so (U= 0.17, [CI: 0.59-1.0] *p*= .86). Further, 4 (12%) subjects reported abstention from alcohol consumption during the quarantine period. More subjects reported an increase (n= 16, 46%) as opposed to a decrease (n= 10, 29%) or no change (n= 9, 26%) of weekly alcohol consumption from November to the quarantine period, although not significantly so (X^2= 0.03, *p*= .85).

Sub-sample analysis by gender

Males

For the males in our sample (n=1,000), the change in problem drinking severity (AUDIT-C) was in 0.91 ± 1.53 [CI: 0.82-1.01] (range: 0-8) and the mean change in the amount consumed was 3.88 ± 8.84 [CI: 3.33-4.42] units per week (range: 0-120). The current problem drinking severity (full AUDIT) was 2.99 ± 4.61 [CI: 2.71-3.28] (range: 0-32), with 403 males included that do not consume alcohol. Of males who reported they consume alcohol (n= 597), the change in severity from pre-quarantine to quarantine was a decrease of -0.4 ± 2.4, [CI: -0.5- -0.21] range -8-8 (U= -3.57, [CI: 0.0-0.03] *p*< .0001). The units of alcohol consumed per week was significantly decreased during the quarantine period (8.52 ± 14 units, [CI: 7.33-9.71] range= 0-120) compared to November (9.23 ± 12.62 units, [CI: 8.21-10.24] range = 0-120), U= -5.2, [CI: 0.0-0.13] *p*< .0001. Further, 128 (20%) males reported abstention from alcohol consumption during the quarantine period. More males reported a decrease (n= 278, 47%) or an increase (n= 204, 34%) as opposed to no change (n= 115, 19%) of weekly alcohol consumption from November to the quarantine period (X^2= 15.94, p < .0001).

Females

For females in our sample (n=342), the change in problem drinking severity (AUDIT-C) was 0.81 ± 1.1 [CI: 0.69-0.92] (range: 0-8) and the mean change in the amount consumed was 2.82 ± 4.6 [CI: 2.31-3.32] units per week (range: 0-38). The current problem drinking severity (full AUDIT) was 3.14 ± 4.47 [CI: 3.13-4] (range: 0-21), with 95 females included that do not consume alcohol. Of females who reported they consume alcohol (n= 247), the change in severity from pre-quarantine to quarantine was an increase of 0.12 ± 1.6, [CI: -0.08-0.32] range -

For peer review only

5-8, although not significantly so (U= 1.17, [CI: 0.01-0.93] *p*= .24). The units of alcohol consumed per week was decreased during the quarantine period (6.94 ± 10.62 units, [CI:] range= 0-80) compared to November (6.01 ± 8.08 units, [CI: 5-7.02] range = 0-90), although not significantly so (U= -0.57, [CI: 0.1-0.99] *p*= .57). Further, 43 (17%) females reported abstention from alcohol consumption during the quarantine period. More females reported a decrease (n= 102, 41%) or an increase (n= 101, 41%) as opposed to no change (n= 44, 18%) of weekly alcohol consumption from November to the quarantine period (X^2= 13.46, *p* = .0002).

For peer review only

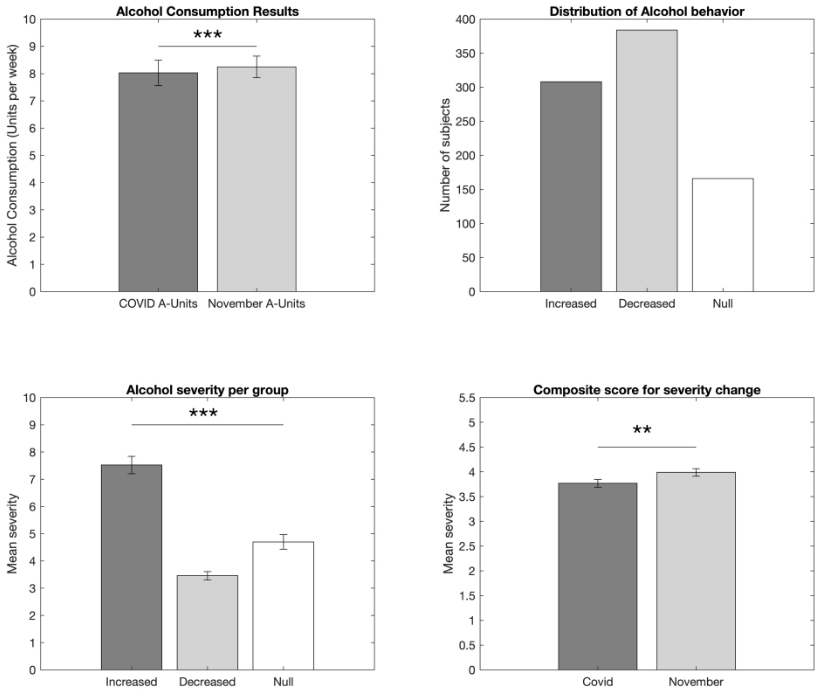

Figure 1. Changes in amount and severity of drinking behaviours in the HabiT sample between pre-quarantine and quarantine periods. Units of alcohol consumed weekly (top left) and changes in drinking severity (AUDIT-C) (bottom right) decreased during the quarantine period and more individuals either increased or decreased their weekly units consumed during quarantine than remained the same (top right). Further, those who increased their weekly alcohol unit consumption during the quarantine period had significantly higher drinking severity indices (full AUDIT) compared to those who decreased or did not change their drinking behaviours during the quarantine period (bottom left).

299x238mm (300 x 300 DPI)

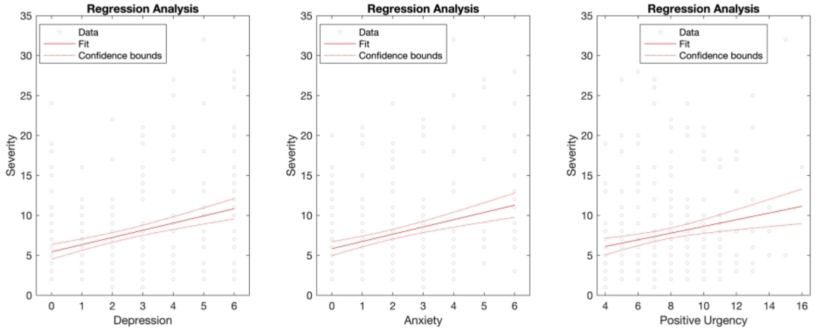

Figure 2. Regression plots of the significant relationships between drinking severity and psychiatric measures in subjects who increased weekly alcohol unit consumption during quarantine. Drinking severity indices of the group who increased their drinking during the quarantine period were significantly positively related to depression severity, anxiety severity, and positive urgency (impulsivity subset).

333x114mm (300 x 300 DPI)

SUPPLEMENTARY MATERIALS

Demographics for drinkers

	Age		Sex		Country		SES		Relationship
Mean	31.4	Male	599	Total	49	Lower	328	Single	449
SD	13.2	Female	248	UK	347	Mid	176	Relationship	408
Range	18-90	Other	12	US	223	Higher	250		
Depression			Anxiety		PTSD		Depression & Anxiety		
41			27		3		35		

Demographic analysis for study dropouts

Although a majority of the dropout subjects (n=1,515) who entered the study provided no data (n=981), we performed a demographic analysis on dropout subjects who provided this information (n=481) to assess if those who completed the survey differed in demographic factors from those who did not. The mean age of dropout subjects was 26.58 ± 11.11 years [CI: 25.59-27.58] (range= 18-80 years), significantly younger than the mean of age of individuals who completed the survey (U= 3.69, [CI: 1.15-3.54] *p*< .0001). Further, more males (n=387) than females (n=87) or other genders (n=7) dropped out of the study prior to completion (X^2= 61.23, *p*< .0001).

Sub-sample analysis by country

United Kingdom (UK)

In the UK, the change in problem drinking severity (AUDIT-C) was 1.05 ± 1.46 [CI: 0.91-1.19] (range: 0-8), and the mean change in the amount consumed was 5.93 ± 11.75 [CI: 4.82-7.05], units per week (range: 0-120). Current problem drinking severity (full AUDIT) was 4.09 ± 4.94 [CI: 3.62-4.56] (range: 0-27). Of the subjects who reported they consume alcohol (n=434), the change in severity from pre-quarantine to quarantine was a decrease of -0.16 ± 2.15, [CI: -0.3-0.06] (range -8-6) but not significantly so (U= -1.38, [CI: 0.01-0.89] *p*= .19). The units of

For peer review only

alcohol consumed per week was significantly increased during the quarantine period (11.25 ± 17.73 units, [CI: 9.36-13.13] range= 1-120) compared to November (10.94 ± 14.17 units, [CI: 9.44-12.45] range = 0-150), U= 3.0, [CI: 0-0.7] p= .003. Further, 60 (14%) subjects reported abstention from alcohol consumption during the quarantine period. More subjects reported a decrease (n= 151, 43%) or an increase (n= 130, 39%) as opposed to no change (n= 61, 18%) of weekly alcohol consumption from November to the quarantine period (X^2= 7.2, p = .007).

United States (US)

In the US, change in problem drinking severity (AUDIT-C) was 1.01 ± 1.55 units [CI: 0.85-1.17] (range: 0-8), and the mean change in the amount consumed was 3 ± 5.51 [CI: 2.39-4] units per week (range: 0-34). The current problem drinking severity (full AUDIT) was 3.48 ± 4.95 [CI: 3-4] (range: 0-32). Of the subjects who reported they consume alcohol (n= 353), the change in severity from pre-quarantine to quarantine was a decrease of -0.11 ± 2.42 [CI: -0.43-0.21], range -8-8 (U= -0.66, [CI: 0.05-0.9] p= .51), but not significantly so. The units of alcohol consumed per week increased between the quarantine period (7.39 ± 11.45 units, [CI: 5.88-8.9] range= 0-80) and November (6.93 ± 9.78 units, [CI: 5.88-8.9] range = 0-96), but not significantly so (U= -1.1, [CI: 0.01-0.94] p= .29). Further, 44 (13%) subjects reported abstention from alcohol consumption during the quarantine period. More subjects reported a decrease (n= 90, 41%) or an increase (n= 88, 40%) as opposed to no change (n= 45, 21%) of weekly alcohol consumption from November to the quarantine period (X^2= 8.15, p = .004).

Canada

In Canada, change in problem drinking severity (AUDIT-C) was 0.67 ± 1.45 [CI: 0.31-1.03] (range: 0-8), and the mean change in the amount consumed was 3.03 ± 7.45 [CI: 1.17-4.89] units per week (range: 0-49). The current problem drinking severity (full AUDIT) was 2.78 ± 4.24 [CI: 1.7-3.85] (range: 0-24). Of the subjects who reported they consume alcohol (n= 35), the change in severity from pre-quarantine to quarantine was an increase of 0.16 ± 2.2, [CI: -0.62-0.95](range= -8-5), but not significantly so (U= .77, [CI: 0.03-0.98] p= .44). The units of alcohol consumed per week was decreased during the quarantine period (8.03 ± 14.22 units, [CI:] range= 0-50) and November (6.71 ± 9.49 units, [CI: 3.46-9.97] range = 0-25), although not

For peer review only - http://bmjopen.bmj.com/site/about/guidelines.xhtml

significantly so (U= 0.17, [CI: 0.59-1.0] *p*= .86). Further, 4 (12%) subjects reported abstention from alcohol consumption during the quarantine period. More subjects reported an increase (n= 16, 46%) as opposed to a decrease (n= 10, 29%) or no change (n= 9, 26%) of weekly alcohol consumption from November to the quarantine period, although not significantly so (X^2= 0.03, *p*= .85).

Sub-sample analysis by gender

Males

For the males in our sample (n=1,000), the change in problem drinking severity (AUDIT-C) was in 0.91 ± 1.53 [CI: 0.82-1.01] (range: 0-8) and the mean change in the amount consumed was 3.88 ± 8.84 [CI: 3.33-4.42] units per week (range: 0-120). The current problem drinking severity (full AUDIT) was 2.99 ± 4.61 [CI: 2.71-3.28] (range: 0-32), with 403 males included that do not consume alcohol. Of males who reported they consume alcohol (n= 597), the change in severity from pre-quarantine to quarantine was a decrease of -0.4 ± 2.4, [CI: -0.5- -0.21] range -8-8 (U= -3.57, [CI: 0.0-0.03] *p*< .0001). The units of alcohol consumed per week was significantly decreased during the quarantine period (8.52 ± 14 units, [CI: 7.33-9.71] range= 0-120) compared to November (9.23 ± 12.62 units, [CI: 8.21-10.24] range = 0-120), U= -5.2, [CI: 0.0-0.13] *p*< .0001. Further, 128 (20%) males reported abstention from alcohol consumption during the quarantine period. More males reported a decrease (n= 278, 47%) or an increase (n= 204, 34%) as opposed to no change (n= 115, 19%) of weekly alcohol consumption from November to the quarantine period (X^2= 15.94, *p* < .0001).

Females

For females in our sample (n=342), the change in problem drinking severity (AUDIT-C) was 0.81 ± 1.1 [CI: 0.69-0.92] (range: 0-8) and the mean change in the amount consumed was 2.82 ± 4.6 [CI: 2.31-3.32] units per week (range: 0-38). The current problem drinking severity (full AUDIT) was 3.14 ± 4.47 [CI: 3.13-4] (range: 0-21), with 95 females included that do not consume alcohol. Of females who reported they consume alcohol (n= 247), the change in severity from pre-quarantine to quarantine was an increase of 0.12 ± 1.6, [CI: -0.08-0.32] range -

For peer review only

5-8, although not significantly so (U= 1.17, [CI: 0.01-0.93] *p*= .24). The units of alcohol consumed per week was decreased during the quarantine period (6.94 ± 10.62 units, [CI:] range= 0-80) compared to November (6.01 ± 8.08 units, [CI: 5-7.02] range = 0-90), although not significantly so (U= -0.57, [CI: 0.1-0.99] *p*= .57). Further, 43 (17%) females reported abstention from alcohol consumption during the quarantine period. More females reported a decrease (n= 102, 41%) or an increase (n= 101, 41%) as opposed to no change (n= 44, 18%) of weekly alcohol consumption from November to the quarantine period (X^2= 13.46, *p* = .0002).

For peer review only

Reporting checklist for cross sectional study.

Based on the STROBE cross sectional guidelines.

Instructions to authors

Complete this checklist by entering the page numbers from your manuscript where readers will find each of the items listed below.

Your article may not currently address all the items on the checklist. Please modify your text to include the missing information. If you are certain that an item does not apply, please write "n/a" and provide a short explanation.

Upload your completed checklist as an extra file when you submit to a journal.

In your methods section, say that you used the STROBE cross sectionalreporting guidelines, and cite them as:

von Elm E, Altman DG, Egger M, Pocock SJ, Gotzsche PC, Vandenbroucke JP. The Strengthening the Reporting of Observational Studies in Epidemiology (STROBE) Statement: guidelines for reporting observational studies.

For peer review only

		Reporting Item	Page Number
**Title and abstract**			
Title	#1a	Indicate the study’s design with a commonly used term in the title or the abstract	1

Abstract	#1b	Provide in the abstract an informative and balanced summary of what was done and what was found	2
**Introduction**			
Background / rationale	#2	Explain the scientific background and rationale for the investigation being reported	4
Objectives	#3	State specific objectives, including any prespecified hypotheses	5
**Methods**			
Study design	#4	Present key elements of study design early in the paper	5
Setting	#5	Describe the setting, locations, and relevant dates, including periods of recruitment, exposure, follow-up, and data collection	5
Eligibility criteria	#6a	Give the eligibility criteria, and the sources and methods of selection of participants.	5
	#7	Clearly define all outcomes, exposures, predictors, potential confounders, and effect modifiers. Give diagnostic criteria, if applicable	6-7
Data sources / measurement	#8	For each variable of interest give sources of data and details of methods of assessment (measurement). Describe comparability of assessment methods if there is more than one group. Give information separately for for exposed and unexposed groups if applicable.	6-7

For peer review only

Bias	#9	Describe any efforts to address potential sources of bias	7-8
Study size	#10	Explain how the study size was arrived at	7-8
Quantitative variables	#11	Explain how quantitative variables were handled in the analyses. If applicable, describe which groupings were chosen, and why	8
Statistical methods	#12a	Describe all statistical methods, including those used to control for confounding	8
Statistical methods	#12b	Describe any methods used to examine subgroups and interactions	8
Statistical methods	#12c	Explain how missing data were addressed	7-8
Statistical methods	#12d	If applicable, describe analytical methods taking account of sampling strategy	N/A
Statistical methods	#12e	Describe any sensitivity analyses	8
**Results**			
Participants	#13a	Report numbers of individuals at each stage of study—eg numbers potentially eligible, examined for eligibility, confirmed eligible, included in the study, completing follow-up, and analysed. Give information separately for for exposed and unexposed groups if applicable.	9
Participants	#13b	Give reasons for non-participation at each stage	8

For peer review only

Participants	#13c	Consider use of a flow diagram	N/A- Cross-sectional survey design
Descriptive data	#14a	Give characteristics of study participants (eg demographic, clinical, social) and information on exposures and potential confounders. Give information separately for exposed and unexposed groups if applicable.	9
Descriptive data	#14b	Indicate number of participants with missing data for each variable of interest	9
Outcome data	#15	Report numbers of outcome events or summary measures. Give information separately for exposed and unexposed groups if applicable.	N/A- survey design
Main results	#16a	Give unadjusted estimates and, if applicable, confounder-adjusted estimates and their precision (eg, 95% confidence interval). Make clear which confounders were adjusted for and why they were included	10
Main results	#16b	Report category boundaries when continuous variables were categorized	10
Main results	#16c	If relevant, consider translating estimates of relative risk into absolute risk for a meaningful time period	N/A no risk

For peer review only

Other analyses	#17	Report other analyses done—e.g., analyses of subgroups and interactions, and sensitivity analyses	9, 12
**Discussion**			
Key results	#18	Summarise key results with reference to study objectives	13-15
Limitations	#19	Discuss limitations of the study, taking into account sources of potential bias or imprecision. Discuss both direction and magnitude of any potential bias.	15-16
Interpretation	#20	Give a cautious overall interpretation considering objectives, limitations, multiplicity of analyses, results from similar studies, and other relevant evidence.	13-15
Generalisability	#21	Discuss the generalisability (external validity) of the study results	16
**Other Information**			
Funding	#22	Give the source of funding and the role of the funders for the present study and, if applicable, for the original study on which the present article is based	16

Notes:

- 13c: N/A- Cross-sectional survey design
- 15: N/A- survey design
- 16c: N/A no risk The STROBE checklist is distributed under the terms of the Creative Commons Attribution License CC-BY. This checklist was completed on 27. August 2020 using

For peer review only

https://www.goodreports.org/, a tool made by the EQUATOR Network in collaboration with Penelope.ai

For peer review only

BMJ Open

Assessing International Alcohol Consumption Patterns During Isolation from the COVID-19 Pandemic Using an Online Survey: Highlighting Negative Emotionality Mechanisms

Journal:	*BMJ Open*
Manuscript ID	bmjopen-2020-044276.R2
Article Type:	Original research
Date Submitted by the Author:	02-Nov-2020
Complete List of Authors:	Sallie, Samantha; University of Cambridge, Psychiatry<br>Ritou, Valentin; University of Paris, Faculty of Basic and Biomedical Sciences<br>Bowden-Jones, Henrietta; University College London, Faculty of Brain Sciences; University of Cambridge, Psychiatry<br>Voon, Valerie; Cambridge University, Psychiatry
**Primary Subject Heading**:	Mental health
Secondary Subject Heading:	Public health, Addiction
Keywords:	COVID-19, PSYCHIATRY, Substance misuse < PSYCHIATRY, PUBLIC HEALTH, Depression & mood disorders < PSYCHIATRY

SCHOLARONE™
Manuscripts

BMJ

I, the Submitting Author has the right to grant and does grant on behalf of all authors of the Work (as defined in the below author licence), an exclusive licence and/or a non-exclusive licence for contributions from authors who are: i) UK Crown employees; ii) where BMJ has agreed a CC-BY licence shall apply, and/or iii) in accordance with the terms applicable for US Federal Government officers or employees acting as part of their official duties; on a worldwide, perpetual, irrevocable, royalty-free basis to BMJ Publishing Group Ltd ("BMJ") its licensees and where the relevant Journal is co-owned by BMJ to the co-owners of the Journal, to publish the Work in this journal and any other BMJ products and to exploit all rights, as set out in our licence.

The Submitting Author accepts and understands that any supply made under these terms is made by BMJ to the Submitting Author unless you are acting as an employee on behalf of your employer or a postgraduate student of an affiliated institution which is paying any applicable article publishing charge ("APC") for Open Access articles. Where the Submitting Author wishes to make the Work available on an Open Access basis (and intends to pay the relevant APC), the terms of reuse of such Open Access shall be governed by a Creative Commons licence – details of these licences and which Creative Commons licence will apply to this Work are set out in our licence referred to above.

Other than as permitted in any relevant BMJ Author's Self Archiving Policies, I confirm this Work has not been accepted for publication elsewhere, is not being considered for publication elsewhere and does not duplicate material already published. I confirm all authors consent to publication of this Work and authorise the granting of this licence.

For peer review only

Assessing International Alcohol Consumption Patterns During Isolation from the COVID-19 Pandemic Using an Online Survey: Highlighting Negative Emotionality Mechanisms

Samantha N. Sallie, MS[a]; Valentin JE. Ritou[a,b]; Henrietta Bowden-Jones, MD[a,c]; Valerie Voon, MD, PhD[a]

[a] Department of Psychiatry, University of Cambridge, Level E4, Addenbrooke's Hospital, Cambridge, United Kingdom, CB2 0QQ.

[b] Faculty of Basic and Biomedical Sciences, University of Paris (Université de Paris), 45 rue des Saints-Pères, Paris, France, 75006.

g

[c] Faculty of Brain Sciences, University College London (UCL), London, United Kingdom, WC1E 6BS.

Corresponding author: Samantha N. Sallie, MS, Department of Psychiatry, University of Cambridge, Level E4, Addenbrooke's Hospital, Cambridge, United Kingdom, CB2 0QQ, sns36@cam.ac.uk

Word Count: 4,618

For peer review only

ABSTRACT

Objectives: The Coronavirus (COVID-19) pandemic has required drastic safety measures to control virus spread, including an extended self-isolation period. Stressful situations increase alcohol craving and consumption in Alcohol Use Disorder (AUD) and non-AUD drinkers. Thus, we assessed how COVID-19-related stress may have affected drinking behaviours in the general population.

Design: We developed an online cross-sectional survey, Habit Tracker (HabiT), which measured changes in drinking behaviours before and during COVID-19 quarantine. We also assessed psychiatric factors such as anxiety, depression (HADS), and impulsivity (SUPPS-P). Lastly, we related drinking behaviours to COVID-19-specific stress factors.

Setting: HabiT was released internationally, with individuals from 83 countries participating.

Participants: Participants were included if they were 18 years of age or older, and confirmed they were proficient in English. The survey was completed by 2,873 adults with 1,346 usable data (46.9% accurately completed).

Primary Outcome Measures: Primary outcome measures were change in amount and severity of drinking behaviours before and during quarantine, and current drinking severity during quarantine.

Results*:* Although drinking behaviors decreased overall during quarantine, 36% reported an increase in alcohol use. Those who increased alcohol use during quarantine were older individuals(CI: 0.04-0.1, *p*<.0001), essential workers(CI: -0.58- -0.1, *p*=.01), individuals with children(CI: -12.46-0.0, *p*=.003), those with a personal relationship with someone severely ill from COVID-19(CI: -2- -0.38, *p*=.01), and those with higher depression(CI: 0.67-1.45, *p*<.0001), anxiety(CI: 0.61-1.5, *p*=.0002), and positive urgency impulsivity(CI: 0.16-0.72, *p*=.009). Further, country-level sub-sample analyses indicated that drinking amount(CI: 9.36-13.13, *p*=.003) increased in the United Kingdom during quarantine.

Conclusions: Our findings highlight a role for identifying those vulnerable for alcohol misuse during periods of self-isolation and underscore the theoretical mechanism of negative emotionality underlying drinking behaviours driven by stress. Limitations include a large degree of study dropout (n=1,515). Future studies should assess the long-term effects of isolation on drinking behaviours.

Keywords: COVID-19; alcohol use; stress; depression; self-isolation

For peer review only

ARTICLE SUMMARY

Strengths and limitations of this study

- The HabiT study sampled drinking behaviours of a large, diverse population during the COVID-19 pandemic.
- Changes in drinking behaviours were assessed against specific COVID-19-related stress factors.
- Due to the length of the survey (8-10 minutes), we observed a large degree of study dropout.
- Subjects were within varying phases of lockdown during the time of testing.
- The prevalence of diagnosed Alcohol Use Disorder drinkers sampled was low, likely related to sampling issues or under-reporting.

For peer review only

INTRODUCTION

The Coronavirus (COVID-19) pandemic has necessitated drastic safety measures to control the virus spread. These measures included an extended self-isolation period in which individuals were permitted to leave their places of residence only to obtain amenities (e.g., food, medical care, toiletries, etc.) or engage in essential work. Individuals were not permitted face-to-face contact with anyone who did not reside within their immediate households. In the United Kingdom, these measures were instituted nationally on March 23rd, 2020, with a gradual lifting of restrictions on May 10th, 2020 ending on July 4th, 2020 with locality-specific intermittent reinstatement of these measures. Although a necessary precautionary measure to mitigate the devastating effects of COVID-19 on public health, evidence indicates that protracted periods of self-isolation, especially in the context of stress, may be related to acute and prolonged negative mental health consequences, particularly in individuals already struggling with psychiatric disorders.[1]

Indeed, current clinical reports from individuals in treatment for Substance Abuse Disorder indicate that the stress produced by COVID-19 social isolation measures have triggered greater and more frequent drug or alcohol cravings, subsequently leading to relapse.[2] This observation is relevant to a prominent mechanistic theory of negative emotionality underlying alcohol misuse.[3] The relationship between stress and alcohol consumption is widely recognised and can be observed in an experimental fashion.[4] In subjects with known Alcohol Use Disorder (AUD), stress and experimental manipulations of stress enhance the amount of alcohol consumed [5, 6], alcohol craving [7], problematic drinking behaviours, and likelihood of relapse.[8] Exposure to stress triggers relapse characterised by a re-instantiation of alcohol cravings and alcohol-seeking behaviours.

Increases in alcohol craving and consumption after stress exposure also occur in those without AUD. An increase in alcohol consumption is often used as a coping strategy for both chronic and specific stressful life events in both AUD and non-AUD drinkers.[9] Similarly in both groups, self-reported craving and subjective judgements of alcohol value rise following a stress task [10], and social drinkers consume more alcohol after witnessing a social stressor.[11] These relationships are moderated by gender [12], age [13], previous alcohol exposure [13], alcohol

For peer review only

expectancies [14], and the pattern of alcohol consumption.[15] Further, psychiatric symptomology such as anxiety and depression as well as pathological levels of personality traits such as impulsivity are widely recognised predisposing factors to problematic alcohol use and addiction.[3, 16]

Thus, in response to these exceptional circumstances, we aimed to assess how social isolation measures in the midst of the COVID-19 pandemic may have affected drinking behaviours in the general adult population. We developed an international survey, entitled Habit Tracker (HabiT), which evaluated drinking severity before (post-hoc recall) and during the COVID-19 quarantine period. We hypothesised that changes in amount of alcohol consumption and severity of drinking behaviours may be related to specific COVID-19 related stress factors, as well as demographic and psychiatric factors. Further, we investigated if COVID-19-related stress factors influenced changes in drinking amount, drinking severity, depression, and anxiety before and during quarantine.

METHODS

Recruitment and inclusion criteria

The HabiT survey was a questionnaire that sought to assess the effects of isolation on alcohol, smoking, and internet use. The effects on alcohol use are reported here. Subjects were included if they were 18 years of age or older and confirmed they were proficient in reading and understanding English. HabiT was advertised by University of Cambridge news page on May 11th, 2020, a day before its international release. For the next several days, the survey was disseminated by news agencies throughout the United Kingdom (e.g., The Telegraph, BBC Cambridgeshire, News Wise) as well as throughout various University of Cambridge colleges. Further, the survey was posted and shared on personal and public social media sites (i.e., Facebook, Twitter). HabiT was approved by the Cambridge Psychology Research Ethics Committee. All subjects gave informed consent and were not financially compensated for their participation, although informed that- upon survey completion- they would be provided results of the study through request. The data collected was fully anonymised. The survey was created using Qualtrics (Provo, Utah) survey-building platform. Developed iteratively within-lab and among co-authors to insure brevity and consistency, the average time to complete the survey was

For peer review only

approximately 8-10 minutes, and all subjects could participate on either a computer or smart phone device.

Patient and public involvement statement

We did not involve patients or the public in the research design, reporting, or survey dissemination strategies of this study.

Demographic information

The demographic information collected were as follows: age, gender, socioeconomic status, intimate relationship status, country and city of residence, and any previous or current diagnosis of a psychiatric or neurological disorder.

Attentional checks

Every major section of the survey contained at least one question which served as an attentional check to ensure subjects were correctly reading and answering survey questions to the best of their ability. The attentional checks were structured to mirror the Likert scaling of each section (e.g., "If you are reading this question, please select 'Strongly Agree.'").

Frequency and severity of alcohol consumption before and during the quarantine period

We first asked subjects if they drank alcohol. If the answer was negative, they proceeded to the next set of questions. If the answer was positive, we assessed the change in the amount and severity of alcohol use as well as the current severity of alcohol use. We asked subjects to report the following behaviours within a typical week in November (i.e. pre-quarantine) and within the last week (i.e. during quarantine): (i) the number of units of alcohol consumed within the last week with examples for the number of units for differing types of alcohol and sizes provided; (ii) the change in severity using a time-scale adaptation of the first three questions of the Alcohol Use Disorders Identification Test (AUDIT-C).[17] Subjects were asked to report how many days in the last week they consumed an alcoholic beverage, how many drinks they consumed on a typical day they were drinking in the last week, and how often they consumed six or more drinks

For peer review only

on one occasion in the last week. To assess the current severity of drinking behaviours during quarantine, we used a timescale-adapted version of the full AUDIT [18] which assessed problem drinking behaviours within the last week such as an inability to stop drinking once started, failure to perform responsibilities, feeling guilt or remorse, drinking shortly after waking to ease the adverse physiological effects of drinking the night before, drinking to the point of memory loss, injuring oneself or others due to drinking, and concern from a loved one or medical professional related to the frequency or severity of one's drinking. We used two primary outcome measures: the change in severity (AUDIT-C) corroborated with the secondary change in amount of drinking (units per week) and current severity (full AUDIT).

COVID-19-related stress factors

We assessed 10 factors which may contribute to COVID-19-related stress using the following questions:

1. Have you been deemed an "essential worker" by your government?
2. Do you work for health care services specifically with individuals who have contracted Coronavirus (COVID-19)? (Sub-question of question 1)
3. Has your employment situation changed due to the Coronavirus (COVID-19) crisis?
4. Has anyone you know personally contracted or have shown symptoms characteristic of Coronavirus (COVID-19)?
5. Has anyone you know personally become severely ill or died due to contracting Coronavirus (COVID-19)?
6. Are you isolated alone?
7. Do you have children?
8. If you have children, are you their only caretaker? (Sub-question of question 7)
9. If you are currently in isolation with others, how would you describe the quality of your relations?
10. How often do you currently go outdoors (for work, essential duties, leisure, etc.)?

For peer review only

Psychiatric measures

Depression and anxiety symptomology were measured using The Hospital Anxiety and Depression Scale (HADS); a brief, validated four-item questionnaire.[19] As a secondary analysis, we assessed impulsivity using the validated Short Impulsive-Behavior Scale (SUPPS-P).[20] This scale provides an overall impulsivity score, as well as five scores corresponding to impulsivity subscales: perseveration, lack of premeditation, sensation-seeking, negative urgency, and positive urgency.

Statistical analysis

Statistical analyses were performed using MATLAB (Version 2020a). All subjects who answered the attentional checks incorrectly (n=12), reported highly improbable answers regarding the units of alcohol they consumed weekly (e.g., 1,000 units), did not report their gender, or did not complete the psychiatric questionnaires were excluded from further analysis, leaving a total of 1346 subjects. Drinking severity scores of the sample were non-normally distributed (Shapiro-Wilk, $p < .05$), thus non-parametric tests were used.

We used Mann-Whitney U-tests to compare weekly alcohol unit consumption and alcohol severity before and during quarantine in the full group. Then, we divided subjects into three groups, those who during quarantine either increased, decreased, or did not change their alcohol consumption and performed a Kruskal-Wallis H-test to assess the relative drinking amount to severity indices of these groups.

We then assessed which COVID-19-related stress factors were associated with changes in either amount (alcohol units consumed per week), change in severity (AUDIT-C), current severity (full AUDIT), or current depression and anxiety using the following tests: 1) Mann-Whitney U-Tests to compare negative versus positive responses to the COVID-19 stress factors (MW), 2) MANCOVA [21] controlling for gender and age (MAN1), and 3) A second MANCOVA controlling for age, gender, depression, and anxiety symptomology (MAN2). For the MANCOVA tests, variables ‘age,’ ‘depression severity,’ and ‘anxiety severity’ were dichotomised via median split. For the COVID-19 stress primary factor comparisons (eight items), we used False Discovery Rate (FDR) to control for multiple comparisons with

For peer review only

significance assigned at $p < .05$.[22, 23] Confidence intervals (CIs) are provided with p values for significant findings observed from the most stringent statistical test.

On an exploratory basis, we then used Spearman's partial correlation to compare the drinking severity indices of subjects who completed the timescale-adapted full AUDIT with SUPPS-P and HADS scores to relate drinking severity of the overall sample to psychiatric measures. Lastly, in order to assess possible directional relationships in changes in the severity of drinking behaviors to depression, anxiety, and impulsivity; we performed Spearman's partial correlations with the psychiatric questionnaires among the three aforementioned groups (i.e., increased, decreased, and null). For both correlational analyses, we used FDR correction ($p < .05$) for multiple comparisons.

RESULTS

Demographic information

A total of 2,873 subjects participated (data collection: 05/12/2020 to 05/28/2020) of which 1,346 had usable data based on defined criteria (1,515 dropouts; 46.9% accurately completed; please refer to the supplementary materials for a demographic analysis of those who did not complete the survey). Of these subjects, 859 (63.8%) reported that they drink alcohol (please refer to the supplementary materials for demographic information for those report drinking alcohol). Of the 1346 subjects, the average age was 28.92 ± 10.45 years [CI: 28.2-29.53] (range= 18-90) with more males (males: n= 1006; females: n=325; other: n=15) from 85 different countries of residence, with the majority from the United Kingdom (n= 434) and the United States (n= 355), followed by Canada (n= 64) and Germany (n= 63). Marital status was as follows: single: n=785; married or committed: n=571; divorced or separated: n=33; widowed: n=4. Socioeconomic status (as denoted by annual income in raw currency on the country-level and converted to UK pounds during analysis) was as follows: <19.9k: n=285; 20-39.9k: n= 273; 20-39.9k: n=244; 40-69.9k: n=241; 70-99.9k: n=141; >100k: n=203; and 232 subjects did not report their incomes. Current psychiatric or neurological diagnoses were as follows: no diagnosis: n=1192; depression: n= 60; anxiety: n= 38; Post-Traumatic Stress Disorder (PTSD): n= 5; comorbid depression and anxiety: n= 46.

For peer review only - http://bmjopen.bmj.com/site/about/guidelines.xhtml

Overall changes in drinking frequency and severity before and during quarantine

Of the total sample, the change in problem drinking severity (AUDIT-C) was 0.89 ± 1.43 [CI: 0.81-0.96] (range: 0-8) and the mean change in the amount consumed was 5.62 ± 9.55 units per week [CI: 3.16-4.02] (range: 0-120). The current problem drinking severity (full AUDIT) was 3.14 ± 4.47 [CI: 2.9-3.37] (range: 0-32), with 557 subjects included that do not consume alcohol. Of the subjects who reported they consume alcohol (n= 859), the change in severity from pre-quarantine to quarantine was a decrease of 1.53 ± 1.6, [CI: 5.01-5.64] range 0-8 (U= 2.65, [CI: 0-0.21] *p*= .008). The units of alcohol consumed per week was significantly decreased during the quarantine period (8.03 ± 14.22 units, [7.11-8.94] range= 1-120) compared to November (8.32 ± 11.92 units, [CI: 7.47-9.02] range = 0-150), U= -2.29, [CI: 0.0-0.0] *p*= .02 (Figure 1). However, in the UK, the units of alcohol consumed per week was significantly increased during the quarantine period (11.25 ± 17.73 units, [CI: 9.36-13.13] range= 1-120) compared to November (10.94 ± 14.17 units, [CI: 9.44-12.45] range = 0-150), U= 3.0, [CI: 0-0.7] *p*= .003. (For full country-level sub-analyses of drinking behaviours, as well as severity of lockdown and amount of confirmed COVID-19 cases and deaths during the data collection period by country via Coronavirus Government Response Tracker [24]; please refer to the supplementary materials). Of the international sample, 172 (20%) subjects reported abstention from alcohol consumption during the quarantine period.. More subjects reported a decrease (n= 384, 45%) or an increase (n= 308, 36%) as opposed to no change (n= 166, 19%) of weekly alcohol consumption from November to the quarantine period (X^2= 72.86, *p*= .001; Figure 1).. Of the three groups, those who: 1) increased weekly units consumed during quarantine (7.5 ± 10.5 change in units, [CI: 6.33-8.7] range: 1-80), 2) decreased weekly units consumed during quarantine (-6.5 ± 9.5 change in units, [CI: -7.45- -5.55] range: -.2 - -120), and 3) did not change their weekly unit consumption, subjects who had increased the units of alcohol consumed during the quarantine period showed significantly higher current drinking severity scores (7.5 ± 5.6, [CI: 6.89-8.15] range: 1-32) than those who reported decreases (3.5 ± 3.0, [CI: 3.16-3.76] range: 1-21) or no changes (4.8 ± 3.6, [CI: 4.17-5.23] range: 1-20) in weekly unit consumption (H= 165.33, [CI: 3.35-4.78] *p* < .0001, Figure 1).

[INSERT FIGURE 1 & FIGURE 1 LEGEND HERE]

For peer review only - http://bmjopen.bmj.com/site/about/guidelines.xhtml

COVID-19 stress factor evaluation

The change in amount of drinking was positively correlated with age (r_s = 0.2, [CI: 0.04-0.1] p < .0001), and gender with males (6.44 ± 10.8 units, [CI: 5.63-7.35] range: 0-120) showing an increased change in drinking amount relative to females (3.81 ± 5.18, [CI: 3.08-4.32] range: 0-38) or other genders (1.32 ± 1.65, [CI: 0.18-2.24] range: 0-5) (H= 8.17, p = .003). Changes in drinking severity were also related to both age and gender, with older individuals (r_s= .2, [CI: 0.01-0.02] p < .0001) and males (1.68 ± 1.74, [CI: 1.55-1.83] range: 0-8) demonstrating greater changes in their drinking severity than females (1.16 ± 1.12, [CI: 1.02-1.3] range: 0-8) and others (1.36 ± 1.29, [CI: 0.54-2.18] range: 0-3) (H= 6.02, [CI: -0.81- -0.22] p = .05). (Gender-specific sub-analyses of drinking behaviours can be found in the supplementary materials). Thus, we utilised age and gender as covariates for both MANCOVA analyses. All relevant covariates used in these analyses were dichotomised via median split (age= 25.1 years, depression severity= 2, and anxiety severity= 1).

Primary COVID-19 stress factors

The influence of COVID-19 stress factors on the change in drinking severity, amounts consumed, and current drinking severity are reported in Tables 1, 2, and 3, respectively. Designated essential workers and those with children showed a greater increase in the amount consumed weekly and drinking severity as well as greater current severity. This remained significant including when controlled for demographic variables (age, gender) and psychiatric symptoms (depression, anxiety). Notably, although subjects with children reported an increase in the number of units of alcohol and severity of alcohol use, they also reported lower levels of depression and anxiety. Knowing an individual personally who was ill or severely ill with Covid-19 showed higher current alcohol drinking severity than those who did not, but with no change from pre- to post-quarantine. A reported change in employment status and isolating alone was associated with greater depression scores, with no differences in drinking behaviours. Isolating with others but reporting a poor relationship was associated with greater depression and anxiety, however, the lower drinking behaviours were moderated by age and gender effects. Finally, going outdoors was associated with greater current drinking severity and greater depression and

anxiety scores controlling for all variables. Post-hoc tests confirmed that, in cases in which a significant relationship was lost between an item and either changes in drinking frequency or severity due to controlling for age and gender (i.e., MANCOVA 1), age was the sole contributor (Essential worker: $F(1, 533.2)= 7$, [CI: 0.15-2.1] $p= .008$; Others ill: $F(1, 879.9)= 52.6$, [CI: 1.7-2.7] $p < .0001$; Poor relationship: $F(1, 933.9)= 48.88$, [CI: 1.8-2.8] $p < .0001$).

Stress Factor	N Total	Yes M(SD)	N Yes	No M(SD)	N No	MW p-value	MAN1 p-value	MAN2 p-value	CI
Essential worker	1337	0.16(1.9)	241	-0.21(1.6)	1096	0.02*	0.01*	0.01*	-0.58- -0.1
Employment	1337	-0.14(1.8)	323	-0.14(1.6)	1014	0.83	0.96	0.92	
Others ill	1334	-0.17(1.8)	497	-0.12(1.6)	837	0.75	0.64	0.63	
Others severely ill	1336	-0.01(2)	127	-0.15(1.6)	1209	0.35	0.7	0.69	
Isolated alone	1325	-0.1(1.9)	168	-0.15(1.6)	1157	0.83	0.85	0.82	
Having children	1334	0.34(1.4)	209	-0.23(1.7)	1125	<.0001*	0.005*	0.003*	-12.46-0.0
Poor relationship	1168	-0.3(1.7)	187	-0.13(1.6)	981	0.35	0.7	0.69	
Going outdoors	1336	-0.27(1.3)	193	-0.12(1.7)	1143	0.26	0.7	0.69	

Table 1. COVID-19 primary stress items relationship with changes in drinking severity (as indexed by the AUDIT-C) from pre-quarantine to quarantine.

Stress Factor	N Total	Yes M(SD)	N Yes	No M(SD)	N No	MW p-value	MAN1 p-value	MAN2 p-value	CI
Essential worker	1337	1.26(12.8)	241	0.45(7.5)	1096	0.0003*	0.07	0.08	-3.4- -0.02
Employment	1337	0.17(11.2)	323	0.13(7.8)	1014	0.77	0.95	0.97	
Others ill	1334	0.05(7.1)	497	0.2(9.6)	837	0.83	0.95	0.97	
Others severely ill	1336	0.06(7.6)	127	0.15(8.9)	1209	0.83	0.95	0.97	
Isolated alone	1325	0.05(11.6)	168	0.2(8.2)	1157	0.46	0.95	0.97	
Having children	1334	2.02(11.9)	209	0.54(7.9)	1125	<.0001*	0.04*	0.02*	-3.6- - 0.74
Poor relationship	1168	0.4(6.1)	187	0.19(8.7)	981	0.46	0.95	0.97	
Going outdoors	1336	1.23(6.8)	193	0.04(9.0)	1143	0.15	0.47	0.4	

Table 2. COVID-19 primary stress items relationship with changes in drinking amount (in units) from pre-quarantine to quarantine.

Stress Factor	N Total	Severity Type	Yes M(SD)	N Yes	N M(SD)	N No	M-W p-value	MAN1 p-value	MAN2 p-value	CI
Essential worker	1337	Drinking	4.42(5.7)	243	2.85(4.1)	1099	<.0001*	0.0005*	0.0005*	-1.8- -057
		Depression	2.29(1.8)	243	2.44(1.9)	1099	0.43	0.84		
		Anxiety	1.79(1.7)	243	1.94(1.8)	1099	0.42	0.8		
Employment change	1337	Drinking	3.46(4.9)	324	3.02(4.3)	1018	0.38	0.08	0.144	
		Depression	2.78(2.0)	324	2.31(1.9)	1018	0.0043*	0.007*		-0.58- -0.1
		Anxiety	2.03(4.5)	324	1.88(1.8)	1018	0.32	0.363		
Others ill	1334	Drinking	3.59(1.9)	499	2.87(4.4)	840	<.0001*	0.1	0.125	-1.2- -0.2
		Depression	2.3(1.8)	499	2.47(1.9)	840	0.20	0.83		
		Anxiety	1.9(5.5)	499	1.93(1.9)	840	0.99	0.94		
Others severely ill	1336	Drinking	4.49(2.0)	127	2.99(4.3)	1214	0.001*	0.007*	0.01*	-2- -0.38
		Depression	2.45(2.0)	127	2.4(1.9)	1214	0.99	0.41		

		Anxiety	1.92(5.8)	127	1.91(1.8)	1214	0.82	0.84		
Isolated alone	1325	Drinking	3.88(2.0)	169	2.98(4.2)	1161	0.42	0.83	0.87	
		Depression	3.4(1.9)	169	2.41(1.9)	1161	0.009*	0.04*		-0.7- -0.06
		Anxiety	2.04(5.2)	169	1.9(1.8)	1161	0.43	0.11		
Having children	1334	Drinking	5.17(1.8)	211	2.75(4.2)	1128	< 0001*	0.0003*	<.0001*	-2.4- -0.9
		Depression	1.5(1.7)	211	2.58(1.9)	1128	<.0001*	<.0001*		0.37-0.97
		Anxiety	1.37(1.7)	211	2.02(1.9)	1128	<.0001*	0.0009*		0.25-0.85
Poor relationship	1168	Drinking	2.82(5.1)	187	3.1(4.1)	985	0.01*	0.92	0.87	0.4- 1.0
		Depression	3.57(2.0)	187	2.2(1.8)	985	<.0001*	<.0001*		-1.53- -1
		Anxiety	2.79(2.0)	187	1.74(1.8)	985	<.0001*	<.0001*		-1.3- -073
Going outdoors	1336	Drinking	3.42(4.5)	1148	1.37(3.4)	193	<.0001*	<.0001*	<.0001*	1.14-2.47
		Depression	3.18(2.0)	193	2.28(1.9)	1148	<.0001*	<.0001*		-1- -0.42
		Anxiety	2.42(2.0)	193	1.83(1.8)	1148	0.0002*	0.0008*		-0.8- -0.24

Table 3. COVID-19 primary stress items relationship with current drinking severity (i.e., full AUDIT), depression, and anxiety from pre-quarantine to quarantine.

Secondary COVID-19 stress factors

Two COVID-19 stress factors were considered secondary as they represented a subset of a primary factor. Working for health care services was associated with a trend towards a greater change in amount of units consumed (F= 3.97 [CI: -6.73- -0.0], *p* = .05) and greater severity of current drinking (F= 7.01, [CI: -3.9- -0.6] *p* = .007) when controlled for all variables. Being the only caretaker for children was also associated with greater change in drinking severity (U= 2.62, [CI: -2.7- -0.9] *p* = .009) and greater change of amount consumed (U= 2.67, [CI: -4.5- -0.8] *p* = .007), but was no longer significant when controlling for age and gender.

Drinking severity during quarantine and correlations with psychiatric measures

Of the individuals who reported drinking alcohol, (n= 769) completed the current drinking severity index (e.g., the adapted-timescale full AUDIT). The severity of drinking behaviours was positively related to depression (r_s= .12, [CI: 0.34-.79] *p*= .004), anxiety (r_s= .12, [CI: 0.3-0.74] *p*= .027), and positive urgency impulsivity (r_s= .12, [CI: 0.14-0.34] *p*= .004), controlled for age and gender. To assess potential directional relationships between current drinking severity during quarantine and psychiatric measures, we correlated depression, anxiety, and impulsivity with the three drinking groups (i.e., increased, decreased, null). Drinking severity scores in the decreased and no change groups were not significantly correlated with any of the psychiatric measures of interest. However, drinking severity of those who increased their units consumed during the

For peer review only - http://bmjopen.bmj.com/site/about/guidelines.xhtml

quarantine period were related to depression (r_s= .30, [CI: 0.67-1.45] $p < .0001$), anxiety (r_s= .23, [CI: 0.61-1.5] p= .0002), and positive urgency (r_s= .17, [CI: 0.16-0.72] p= .009) (Figure 2).

[INSERT FIGURE 2 & FIGURE 2 LEGEND HERE]

DISCUSSION

We show an overall decrease in amounts and severity of problem alcohol use from pre-quarantine to the quarantine period. Critically, however, three different subpopulations were identified with most either increasing or decreasing use as compared to remaining unchanged in their alcohol use behaviours. Greater drinking was associated with demographic factors including age and male gender, COVID-19 stress-related factors, and psychiatric factors such as depression, anxiety, or the impulsivity subscale of positive urgency. Our findings underscore the theoretical mechanism of negative emotionality underlying drinking behaviours driven by stress, depression, and anxiety.

An overall decrease in alcohol use and problematic use may have multiple potential etiologies. Stringent lockdown may be associated with a decrease in the presence or availability of alcoholic beverages within the immediate household given limitations in access, a decrease in exposure to alcohol cues that may trigger urges, or the preference to consume alcohol within social contexts. More subjects reported either decreasing or increasing the frequency of their alcohol intake as compared to remaining unchanged, consistent with previous reports of a greater tendency toward extremes in individual drinking patterns when faced with either acute or chronic life stressors.[15]

Older individuals showed a greater increase in drinking behaviours during lockdown and current severity of problem drinking consistent with demographic factors known to be associated with alcohol misuse. Whether one increases their drinking after experiencing acute or chronic life stress is age-dependent, which may reflect a function of previous alcohol experience.[13] Age may play a particularly unique role in the context of COVID-19 due to the greater need for stringent isolation with age, potentially fewer supports, and the risk of greater isolation, loneliness, and concern about the impact of COVID-19 on one's personal health. Expectedly,

males showed greater unit consumption compared to females and other genders overall. However, males showed a decrease in both drinking amount and severity during quarantine, while females demonstrated the opposite trend. This finding corroborates evidence which indicates females are more likely than males to consume alcohol in order to cope with stress.[25]

COVID-19 specific stress factors appear to influence drinking behaviours controlled for other confounding variables. Being deemed an essential worker and having children was associated with a greater increase in drinking behaviours during quarantine. Importantly, although having children was associated with an increase in alcohol use, depression and anxiety scores were lower than in those without children. This suggests the additional burden of childcare and home schooling contributed to the tendency towards drinking possibly in the context of stress relief and was not mediated by greater depression or anxiety symptoms. The presence of children may also be protective against depressive and anxiety symptoms during lockdown. Having children may mitigate against loneliness that has been highlighted as a major issue during the isolation of lockdown.[26] A subset of the essential worker category – health care workers responsible for taking care of individuals with COVID-19 – was associated with greater severity of problem drinking behaviours. Thus, the specific impact of lockdown on the necessity for essential workers and the impact of the burden of home schooling and childcare on parents appears to enhance drinking behaviours independent of an impact on psychiatric symptomatology.

As expected, having a personal relationship with someone who had become severely ill or died due to COVID-19 was associated with a greater increase in severity of problem drinking behaviours. Going outdoors more frequently for work, exercise, or essential duties during lockdown was similarly associated with greater severity of alcohol use, as well as depressive and anxiety symptoms. The reasons behind the need to go outdoors complicate the interpretation, as it might be confounded by being an essential worker but also allow for greater access to the purchase of alcohol. Living with others but having a poor quality of relationship was unexpectedly associated with a lower drinking severity but with greater depressive and anxiety symptoms. Living alone was not associated with any changes in drinking behaviours but was associated with greater depressive symptomatology. These findings might support the role of

For peer review only

drinking in the context of social interactions; and further highlight the importance of social interactions during lockdown, the role of loneliness, and its impact on mental health.[26] Importantly, those residing in the UK- unlike those in the US and Canada- displayed an increase in weekly alcohol units consumed during quarantine, consistent with the WHO Global Status Report on Alcohol and Health (2018) which shows that total alcohol per capita consumption (APC) is higher in the UK than in the US or Canada.[27]

We further observed a relationship between the current severity of drinking behaviours and psychiatric symptoms such as depression, anxiety or positive urgency. These relationships were driven particularly by the group which increased their drinking during quarantine. That both negative and positive emotionality factors are associated with increased drinking behaviours is in keeping with the multiple paths towards alcohol use. The effects of depression and anxiety on alcohol consumption in both AUD and non-AUD drinkers are well-documented [28-31] and related to mechanistic theories of negative emotionality, which suggest that individuals may increase their alcohol consumption in stressful contexts to cope with aversive emotional states.[32] Positive emotional factors appear to also play a role in the association with positive urgency, a subtype of impulsivity characterised by the propensity to engage in disinhibited behaviors including alcohol consumption when experiencing an intensified hedonic or excited state.[31] Positive affect-based impulsivity may reflect a heightened reward sensitivity associated with problem drinking behaviours.[33]

Limitations and future directions

This study is not without limitations. HabiT is a cross-sectional, retrospective survey and hence potentially limited by recall and misclassification biases as well as lack of longitudinal follow-up. Because retrospective reporting involves issues with memory, possible Dunning-Kruger effects, and selection bias; the reader should be cautious in drawing causal interpretations from the current data. Because the aim of the HabiT study was to investigate changes in frequency and severity of drinking behaviour in a large, wider population, we issued the survey internationally and during a later period of enforced isolation. Thus, the possibility cannot be overlooked that subjects were within varying phases of lockdown characterised by differential restrictions during

For peer review only

the time of testing which may have influenced our current results. Future studies may consider data analysis by country, level of lockdown, or amount and severity of localised COVID-19 cases. Also, approximately half of the individuals who began the survey did not complete it. This may be due to the length of the survey (i.e., 8-10 minutes). Prospective studies using an online survey design should further condense questionnaires and/or offer subjects monetary incentives obtained upon survey completion in order to attenuate dropout and non-response bias. The current HabiT survey only assessed the *acute* effects of COVID-19 isolation measures on changes in drinking behaviours in comparison to the pre-quarantine period. Hence, follow-up studies should be employed during the immediate post-quarantine period to investigate the possible protracted effects of COVID-19 isolation on drinking behaviours. Furthermore, whether the sampling adequately reflects the population distribution in the form of sampling bias may be an issue with online questionnaires and may under-represent those who do not have smartphones or access to the internet [34], have limited facility with online questionnaires (e.g., older individuals) [34], were otherwise engaged (e.g., caring for an ill individual or children), or are more severely ill with substance use or other mental health disorders. Thus, our ability to generalise our current findings to the wider population is limited. Other methods (e.g., phone surveys) are recommended to reach populations under-represented by online surveys.[35] As few respondents reported a previous history of alcohol problems relative to the expected prevalence rates, the reporting is likely either a function of sampling bias, limited willingness to reveal such a history in an online survey, or marked changes in alcohol use particularly if relapse occurs. This limits our capacity to assess the change in drinking behaviours in those with a history of alcohol problems. Further studies focusing specifically on the newly abstinent or those with a history of alcohol problems are indicated.

CONCLUSION

Although alcohol drinking behaviours appeared to decrease overall during lockdown, we emphasise that specific groups may be at higher risk for developing problematic alcohol use behaviours. In particular, factors associated with an increase in alcohol use include older individuals, essential workers, parents with children, those with a personal relationship with someone severely ill from COVID-19, and those with higher depression, anxiety levels, or

For peer review only

positive urgency impulsivity. Further, unlike residents from the US and Canada, those in the UK increased their weekly alcohol intake during the quarantine period. We emphasise that those with a previous history of alcohol misuse or a family history of AUD were not the specific focus of this study and may represent a high risk group which requires further investigation. Alcohol can be used in brief, moderate amounts in a healthy, non-pathological manner related to socialisation and stress relief. However, a subgroup of these individuals may still be at higher risk for longer term issues with alcohol misuse. The lockdown resulted in a unique set of stressors that in some cases may persist (e.g. childcare, grieving, prolonged depression or anxiety related to the lockdown) and might again re-emerge with the imposition of localised lockdowns or further lockdowns in the context of a second wave. Further studies on the longitudinal impact and persistence of these behaviours are critical. Our findings highlight a need for identifying those at greater risk for alcohol misuse to aim for greater support services and proactively target mental health issues associated with problem drinking behaviours such as depression or anxiety.

Funding Statement: This research was registered as a no-cost project, under grant number G107438. VV is supported by a MRC Senior Clinical Fellowship (MR/P008747/1).

Conflict of Interest Statement: All authors reported no biomedical financial interests or potential conflicts of interest.

Author Contributions: SS created the HabiT survey, collaborated with VR in analysing the collected data, and drafted and edited the manuscript. VR coded and analysed the data. HBJ collaborated with VV in conceptualising the study. VV conceptualised the study, gave crucial guidance in creating the HabiT survey, and edited the manuscript.

Data Statement: All participant data used in this research is deidentified. Participant data and MATLAB statistical code used for analysis is available upon reasonable request from corresponding author, Samantha N. Sallie, at habittstudy2020@gmail.com.

For peer review only

LEGENDS FOR FIGURES

Figure 1. Changes in amount and severity of drinking behaviours in the HabiT sample between pre-quarantine and quarantine periods. Units of alcohol consumed weekly (top left) and changes in drinking severity (AUDIT-C) (bottom right) decreased during the quarantine period and more individuals either increased or decreased their weekly units consumed during quarantine than remained the same (top right). Further, those who increased their weekly alcohol unit consumption during the quarantine period had significantly higher drinking severity indices (full AUDIT) compared to those who decreased or did not change their drinking behaviours during the quarantine period (bottom left).

Figure 2. Regression plots of the significant relationships between drinking severity and psychiatric measures in subjects who increased weekly alcohol unit consumption during quarantine. Drinking severity indices of the group who increased their drinking during the quarantine period were significantly positively related to depression severity, anxiety severity, and positive urgency (impulsivity subset).

For peer review only

REFERENCES

1 Holmes EA, O'Connor RC, Perry VH, et al. Multidisciplinary research priorities for the COVID-19 pandemic: a call for action for mental health science. *The Lancet Psychiatry*. 2020 Jun 1;7(6):547–60.

2 Volkow ND. Collision of the COVID-19 and addiction epidemics. *Annals of Internal Medicine*. 2020 Apr 2;173(1):61–2.

3 Koob GF. Stress, Corticotropin-releasing factor and drug addiction. *Annals of the New York Academy of Sciences*. 1999;897(1):27–45.

4 McGrath E, Jones A, Field M. Acute stress increases ad-libitum alcohol consumption in heavy drinkers, but not through impaired inhibitory control. *Psychopharmacology*. 2016 Apr 1;233(7):1227–34.

5 Marlatt GA. Taxonomy of high-risk situations for alcohol relapse: evolution and development of a. *Addiction*. 1996;91(12s1):37–50.

6 Amlung M, MacKillop J. Understanding the effects of stress and alcohol cues on motivation for alcohol via behavioral economics. *Alcoholism: Clinical and Experimental Research*. 2014;38(6):1780–9.

7 Fox HC, Bergquist KL, Hong K-I, et al. Stress-induced and alcohol cue-induced craving in recently abstinent alcohol-dependent individuals. *Alcoholism: Clinical and Experimental Research*. 2007;31(3):395–403.

8 Noone M, Dua J, Markham R. Stress, cognitive factors, and coping resources as predictors of relapse in alcoholics. *Addictive Behaviors*. 1999 Sep 1;24(5):687–93.

9 Cooper ML, Russell M, George WH. Coping, expectancies, and alcohol abuse: A test of social learning formulations. *Journal of Abnormal Psychology*. 1988;97(2):218–30.

For peer review only

10 Owens MM, Ray LA, MacKillop J. Behavioral economic analysis of stress effects on acute motivation for alcohol. *Journal of the Experimental Analysis of Behavior*. 2015;103(1):77–86.

11 Magrys SA, Olmstead MC. Acute stress increases voluntary consumption of alcohol in undergraduates. *Alcohol*. 2015 Mar 1;50(2):213–8.

12 Wilsnack RW, Wilsnack SC, Kristjanson AF, Vogeltanz-Holm ND, Gmel G. Gender and alcohol consumption: Patterns from the multinational genacis project. *Addiction*. 2009 Sep;104(9):1487–500.

13 Spanagel R, Noori HR, Heilig M. Stress and alcohol interactions: Animal studies and clinical significance. *Trends in Neurosciences*. 2014 Apr 1;37(4):219–27.

14 Clay JM, Adams C, Archer P, English M, et al. Psychosocial stress increases craving for alcohol in social drinkers: Effects of risk-taking. *Drug and Alcohol Dependence*. 2018 Apr 1;185:192–7.

1.

15 José BS, Van Oers H a. M, Van De Mheen HD, Garretsen HFL, Mackenbach JP. Stressors and alcohol consumption. *Alcohol*. 2000 May 1;35(3):307–12.

16 Dick DM, Smith G, Olausson P, Mitchell SH, Leeman RF, O'Malley SS, et al. Understanding the construct of impulsivity and its relationship to alcohol use disorders. Addict Biol. 2010 Apr;15(2):217–26.

17 Bush K, Kivlahan DR, McDonell MB, Fihn SD, et al. The AUDIT alcohol consumption questions (AUDIT-C): An effective brief screening test for problem drinking. *Arch Intern Med*. 1998 Sep 14;158(16):1789–95.

For peer review only

18 Saunders JB, Aasland OG, Babor TF, et al. Development of the alcohol use disorders identification test (AUDIT): WHO collaborative project on early detection of persons with harmful alcohol consumption-II. *Addiction*. 1993;88(6):791–804.

19 Snaith RP. The hospital anxiety and depression scale. *Health Qual Life Outcomes*. 2003 Aug 1;1:29.

20 Cyders MA, Littlefield AK, Coffey S, et al. Examination of a short English version of the UPPS-P impulsive behavior scale. *Addictive Behaviors*. 2014 Sep 1;39(9):1372–6.

21 MANCOVAN [Internet]. [cited 2020 Aug 13]. Available from: https://uk.mathworks.com/matlabcentral/fileexchange/27014-mancovan

22 Benjamini Y, Hochberg Y. Controlling the false discovery rate: A practical and powerful approach to multiple testing. *Journal of the Royal Statistical Society*: Series B (Methodological). 1995 Jan 1;57(1):289–300.

23 fdr_bh [Internet]. [cited 2020 Aug 13]. Available from: https://uk.mathworks.com/matlabcentral/fileexchange/27418-fdr_bh

24 Hale T, Webster S, Petherick A, Philips T, Kira B. Oxford COVID-19 government response tracker, Blavatnik School of Government (2020). Data use policy: Creative Commons Attribution CC BY standard.

25 Peltier MR, Verplaetse TL, Mineur YS, Petrakis IL, Cosgrove KP, Picciotto MR, et al. Sex differences in stress-related alcohol use. Neurobiology of Stress. 2019 Feb 1;10:100149.

26 Banerjee D, Rai M. Social isolation in Covid-19: The impact of loneliness. *Int J Soc Psychiatry*. 2020 Sep 1;66(6):525–7.

For peer review only

27 Global status report on alcohol and health 2018. Geneva: World Health Organization; 2018. Licence: CC BY-NC-SA 3.0 IGO

28 Peirce RS, Frone MR, Russell M, et al. A longitudinal model of social contact, social support, depression, and alcohol use. *Health Psychology*. 2000;19(1):28–38.

29 Battista S, Stewart S, Ham L. A critical review of laboratory-based studies examining the relationships of social anxiety and alcohol intake. *CDAR*. 2010 Mar 1;3(1):3–22.

30 Smith JP, Randall CL. Anxiety and alcohol use disorders. *Alcohol Res*. 2012;34(4):414–31.

31 Brière FN, Rohde P, Seeley JR, et al. Comorbidity between major depression and alcohol use disorder from adolescence to adulthood. *Comprehensive Psychiatry*. 2014 Apr 1;55(3):526–33.

32 Cyders MA, Smith GT. Mood-based rash action and its components: Positive and negative urgency. *Personality and Individual Differences*. 2007 Sep 1;43(4):839–50.

33 Dinc L, Cooper AJ. Positive affective states and alcohol consumption: The moderating role of trait positive urgency. *Addictive Behaviors*. 2015 Aug 1;47:17–21.

34 Remillard ML, Mazor KM, Cutrona SL, et al. Systematic review of the use of online questionnaires among the geriatric population. *J Am Geriatr Soc*. 2014 Apr;62(4):696–705.

35 Szolnoki G, Hoffmann D. Online, face-to-face and telephone surveys—Comparing different sampling methods in wine consumer research. *Wine Economics and Policy*. 2013 Dec 1;2(2):57–66.

For peer review only

For peer review only

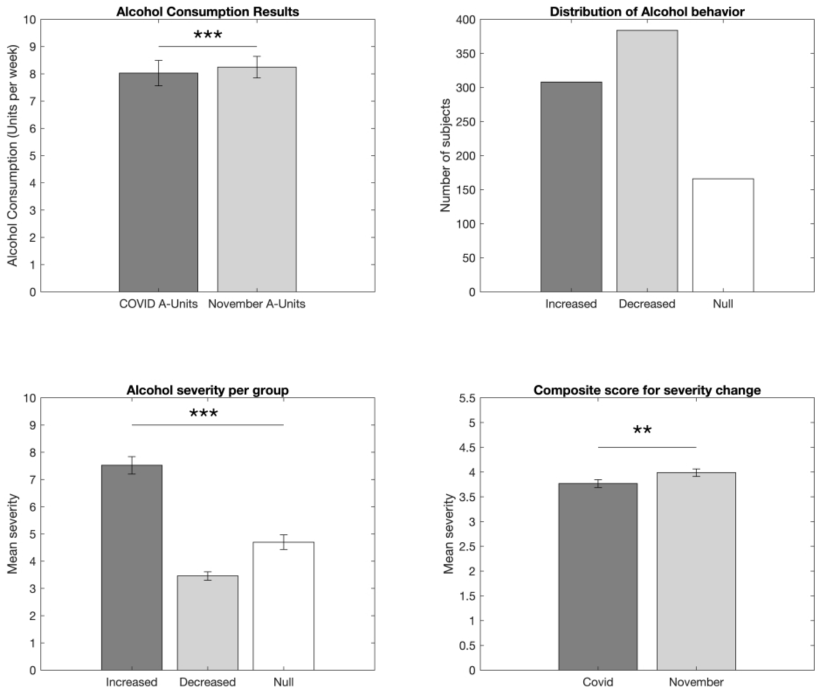

Figure 1. Changes in amount and severity of drinking behaviours in the HabiT sample between pre-quarantine and quarantine periods. Units of alcohol consumed weekly (top left) and changes in drinking severity (AUDIT-C) (bottom right) decreased during the quarantine period and more individuals either increased or decreased their weekly units consumed during quarantine than remained the same (top right). Further, those who increased their weekly alcohol unit consumption during the quarantine period had significantly higher drinking severity indices (full AUDIT) compared to those who decreased or did not change their drinking behaviours during the quarantine period (bottom left).

299x238mm (600 x 600 DPI)

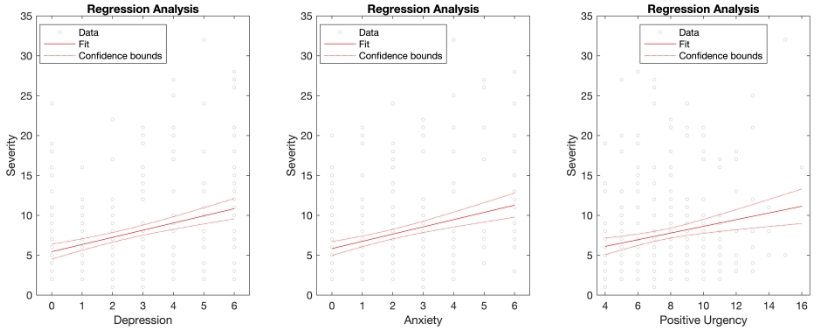

Figure 2. Regression plots of the significant relationships between drinking severity and psychiatric measures in subjects who increased weekly alcohol unit consumption during quarantine. Drinking severity indices of the group who increased their drinking during the quarantine period were significantly positively related to depression severity, anxiety severity, and positive urgency (impulsivity subset).

333x114mm (600 x 600 DPI)

SUPPLEMENTARY MATERIALS

Demographics for drinkers

	Age		Sex		Country		SES		Relationship
Mean	31.4	Male	599	Total	49	Lower	328	Single	449
SD	13.2	Female	248	UK	347	Mid	176	Relationship	408
Range	18-90	Other	12	US	223	Higher	250		

Depression	Anxiety	PTSD	Depression & Anxiety
41	27	3	35

Demographic analysis for study dropouts

Although a majority of the dropout subjects (n=1,515) who entered the study provided no data (n=981), we performed a demographic analysis on dropout subjects who provided this information (n=481) to assess if those who completed the survey differed in demographic factors from those who did not. The mean age of dropout subjects was 26.58 ± 11.11 years [CI: 25.59-27.58] (range= 18-80 years), significantly younger than the mean of age of individuals who completed the survey (U= 3.69, [CI: 1.15-3.54] $p< .0001$). Further, more males (n=387) than females (n=87) or other genders (n=7) dropped out of the study prior to completion ($X^2= 61.23$, $p< .0001$).

Sub-sample analysis by country

United Kingdom (UK)

In the UK, the change in problem drinking severity (AUDIT-C) was 1.05 ± 1.46 [CI: 0.91-1.19] (range: 0-8), and the mean change in the amount consumed was 5.93 ± 11.75 [CI: 4.82-7.05], units per week (range: 0-120). Current problem drinking severity (full AUDIT) was 4.09 ± 4.94 [CI: 3.62-4.56] (range: 0-27). Of the subjects who reported they consume alcohol (n=434), the change in severity from pre-quarantine to quarantine was a decrease of -0.16 ± 2.15, [CI: -0.3-0.06] (range -8-6) but not significantly so (U= -1.38, [CI: 0.01-0.89] $p= .19$). The units of

For peer review only

alcohol consumed per week was significantly increased during the quarantine period (11.25 ± 17.73 units, [CI: 9.36-13.13] range= 1-120) compared to November (10.94 ± 14.17 units, [CI: 9.44-12.45] range = 0-150), U= 3.0, [CI: 0-0.7] p= .003. Further, 60 (14%) subjects reported abstention from alcohol consumption during the quarantine period. More subjects reported a decrease (n= 151, 43%) or an increase (n= 130, 39%) as opposed to no change (n= 61, 18%) of weekly alcohol consumption from November to the quarantine period (X^2= 7.2, p = .007). The Oxford COVID-19 Government Response Tracker [24] at the country level indicated that the lockdown stringency index in the UK during data collection (05/12/2020 to 05/28/2020) was 88.89, with 15,684 confirmed cases and 488 deaths.

United States (US)

In the US, change in problem drinking severity (AUDIT-C) was 1.01 ± 1.55 units [CI: 0.85-1.17] (range: 0-8), and the mean change in the amount consumed was 3 ± 5.51 [CI: 2.39-4] units per week (range: 0-34). The current problem drinking severity (full AUDIT) was 3.48 ± 4.95 [CI: 3-4] (range: 0-32). Of the subjects who reported they consume alcohol (n= 353), the change in severity from pre-quarantine to quarantine was a decrease of -0.11 ± 2.42 [CI: -0.43-0.21], range -8-8 (U= -0.66, [CI: 0.05-0.9] p= .51), but not significantly so. The units of alcohol consumed per week increased between the quarantine period (7.39 ± 11.45 units, [CI: 5.88-8.9] range= 0-80) and November (6.93 ± 9.78 units, [CI: 5.88-8.9] range = 0-96), but not significantly so (U= -1.1, [CI: 0.01-0.94] p= .29). Further, 44 (13%) subjects reported abstention from alcohol consumption during the quarantine period. More subjects reported a decrease (n= 90, 41%) or an increase (n= 88, 40%) as opposed to no change (n= 45, 21%) of weekly alcohol consumption from November to the quarantine period (X^2= 8.15, p = .004). The Oxford COVID-19 Government Response Tracker [24] at the country level indicated that the lockdown stringency index in the US during data collection (05/12/2020 to 05/28/2020) was 70.92, with 1,347,916 confirmed cases and 80,684 deaths.

Canada

In Canada, change in problem drinking severity (AUDIT-C) was 0.67 ± 1.45 [CI: 0.31-1.03] (range: 0-8), and the mean change in the amount consumed was 3.03 ± 7.45 [CI: 1.17-4.89] units

per week (range: 0-49). The current problem drinking severity (full AUDIT) was 2.78 ± 4.24 [CI: 1.7-3.85] (range: 0-24). Of the subjects who reported they consume alcohol (n= 35), the change in severity from pre-quarantine to quarantine was an increase of 0.16 ± 2.2, [CI: -0.62-0.95](range= -8-5), but not significantly so (U= .77, [CI: 0.03-0.98] p= .44). The units of alcohol consumed per week was decreased during the quarantine period (8.03 ± 14.22 units, [CI:] range= 0-50) and November (6.71 ± 9.49 units, [CI: 3.46-9.97] range = 0-25), although not significantly so (U= 0.17, [CI: 0.59-1.0] p= .86). Further, 4 (12%) subjects reported abstention from alcohol consumption during the quarantine period. More subjects reported an increase (n= 16, 46%) as opposed to a decrease (n= 10, 29%) or no change (n= 9, 26%) of weekly alcohol consumption from November to the quarantine period, although not significantly so (X^2= 0.03, p= .85). The Oxford COVID-19 Government Response Tracker [24] at the country level indicated that the lockdown stringency index in Canada during data collection (05/12/2020 to 05/28/2020) was 70.83, with 69,981 confirmed cases and 4,993 deaths.

Sub-sample analysis by gender

Males

For the males in our sample (n=1,000), the change in problem drinking severity (AUDIT-C) was in 0.91 ± 1.53 [CI: 0.82-1.01] (range: 0-8) and the mean change in the amount consumed was 3.88 ± 8.84 [CI: 3.33-4.42] units per week (range: 0-120). The current problem drinking severity (full AUDIT) was 2.99 ± 4.61 [CI: 2.71-3.28] (range: 0-32), with 403 males included that do not consume alcohol. Of males who reported they consume alcohol (n= 597), the change in severity from pre-quarantine to quarantine was a decrease of -0.4 ± 2.4, [CI: -0.5- -0.21] range -8-8 (U= -3.57, [CI: 0.0-0.03] p< .0001). The units of alcohol consumed per week was significantly decreased during the quarantine period (8.52 ± 14 units, [CI: 7.33-9.71] range= 0-120) compared to November (9.23 ± 12.62 units, [CI: 8.21-10.24] range = 0-120), U= -5.2, [CI: 0.0-0.13] p< .0001. Further, 128 (20%) males reported abstention from alcohol consumption during the quarantine period. More males reported a decrease (n= 278, 47%) or an increase (n= 204, 34%) as opposed to no change (n= 115, 19%) of weekly alcohol consumption from November to the quarantine period (X^2= 15.94, p < .0001).

Females

For peer review only

For females in our sample (n=342), the change in problem drinking severity (AUDIT-C) was 0.81 ± 1.1 [CI: 0.69-0.92] (range: 0-8) and the mean change in the amount consumed was 2.82 ± 4.6 [CI: 2.31-3.32] units per week (range: 0-38). The current problem drinking severity (full AUDIT) was 3.14 ± 4.47 [CI: 3.13-4] (range: 0-21), with 95 females included that do not consume alcohol. Of females who reported they consume alcohol (n= 247), the change in severity from pre-quarantine to quarantine was an increase of 0.12 ± 1.6, [CI: -0.08-0.32] range -5-8, although not significantly so (U= 1.17, [CI: 0.01-0.93] *p*= .24). The units of alcohol consumed per week was decreased during the quarantine period (6.94 ± 10.62 units, [CI:] range= 0-80) compared to November (6.01 ± 8.08 units, [CI: 5-7.02] range = 0-90), although not significantly so (U= -0.57, [CI: 0.1-0.99] *p*= .57). Further, 43 (17%) females reported abstention from alcohol consumption during the quarantine period. More females reported a decrease (n= 102, 41%) or an increase (n= 101, 41%) as opposed to no change (n= 44, 18%) of weekly alcohol consumption from November to the quarantine period (X^2= 13.46, *p* = .0002).

Reporting checklist for cross sectional study.

Based on the STROBE cross sectional guidelines.

Instructions to authors

Complete this checklist by entering the page numbers from your manuscript where readers will find each of the items listed below.

Your article may not currently address all the items on the checklist. Please modify your text to include the missing information. If you are certain that an item does not apply, please write "n/a" and provide a short explanation.

Upload your completed checklist as an extra file when you submit to a journal.

In your methods section, say that you used the STROBE cross sectionalreporting guidelines, and cite them as:

von Elm E, Altman DG, Egger M, Pocock SJ, Gotzsche PC, Vandenbroucke JP. The Strengthening the Reporting of Observational Studies in Epidemiology (STROBE) Statement: guidelines for reporting observational studies.

For peer review only

		Reporting Item	Page Number
**Title and abstract**			
Title	#1a	Indicate the study’s design with a commonly used term in the title or the abstract	1

Abstract	#1b	Provide in the abstract an informative and balanced summary of what was done and what was found	2
**Introduction**			
Background / rationale	#2	Explain the scientific background and rationale for the investigation being reported	4
Objectives	#3	State specific objectives, including any prespecified hypotheses	5
**Methods**			
Study design	#4	Present key elements of study design early in the paper	5
Setting	#5	Describe the setting, locations, and relevant dates, including periods of recruitment, exposure, follow-up, and data collection	5
Eligibility criteria	#6a	Give the eligibility criteria, and the sources and methods of selection of participants.	5
	#7	Clearly define all outcomes, exposures, predictors, potential confounders, and effect modifiers. Give diagnostic criteria, if applicable	6-7
Data sources / measurement	#8	For each variable of interest give sources of data and details of methods of assessment (measurement). Describe comparability of assessment methods if there is more than one group. Give information separately for for exposed and unexposed groups if applicable.	6-7

For peer review only

Bias	#9	Describe any efforts to address potential sources of bias	7-8
Study size	#10	Explain how the study size was arrived at	7-8
Quantitative variables	#11	Explain how quantitative variables were handled in the analyses. If applicable, describe which groupings were chosen, and why	8
Statistical methods	#12a	Describe all statistical methods, including those used to control for confounding	8
Statistical methods	#12b	Describe any methods used to examine subgroups and interactions	8
Statistical methods	#12c	Explain how missing data were addressed	7-8
Statistical methods	#12d	If applicable, describe analytical methods taking account of sampling strategy	N/A
Statistical methods	#12e	Describe any sensitivity analyses	8
**Results**			
Participants	#13a	Report numbers of individuals at each stage of study—eg numbers potentially eligible, examined for eligibility, confirmed eligible, included in the study, completing follow-up, and analysed. Give information separately for for exposed and unexposed groups if applicable.	9
Participants	#13b	Give reasons for non-participation at each stage	8

For peer review only

Participants	#13c	Consider use of a flow diagram	N/A- Cross-sectional survey design
Descriptive data	#14a	Give characteristics of study participants (eg demographic, clinical, social) and information on exposures and potential confounders. Give information separately for exposed and unexposed groups if applicable.	9
Descriptive data	#14b	Indicate number of participants with missing data for each variable of interest	9
Outcome data	#15	Report numbers of outcome events or summary measures. Give information separately for exposed and unexposed groups if applicable.	N/A- survey design
Main results	#16a	Give unadjusted estimates and, if applicable, confounder-adjusted estimates and their precision (eg, 95% confidence interval). Make clear which confounders were adjusted for and why they were included	10
Main results	#16b	Report category boundaries when continuous variables were categorized	10
Main results	#16c	If relevant, consider translating estimates of relative risk into absolute risk for a meaningful time period	N/A no risk

For peer review only

Other analyses	#17	Report other analyses done—e.g., analyses of subgroups and interactions, and sensitivity analyses	9, 12
**Discussion**			
Key results	#18	Summarise key results with reference to study objectives	13-15
Limitations	#19	Discuss limitations of the study, taking into account sources of potential bias or imprecision. Discuss both direction and magnitude of any potential bias.	15-16
Interpretation	#20	Give a cautious overall interpretation considering objectives, limitations, multiplicity of analyses, results from similar studies, and other relevant evidence.	13-15
Generalisability	#21	Discuss the generalisability (external validity) of the study results	16
**Other Information**			
Funding	#22	Give the source of funding and the role of the funders for the present study and, if applicable, for the original study on which the present article is based	16

Notes:

- 13c: N/A- Cross-sectional survey design
- 15: N/A- survey design
- 16c: N/A no risk The STROBE checklist is distributed under the terms of the Creative Commons Attribution License CC-BY. This checklist was completed on 27. August 2020 using

For peer review only

https://www.goodreports.org/, a tool made by the EQUATOR Network in collaboration with Penelope.ai

For peer review only
